# DRaCOon: a novel algorithm for pathway-level differential co-expression analysis in transcriptomics

**DOI:** 10.1186/s12859-025-06162-9

**Published:** 2025-05-26

**Authors:** Fernando M. Delgado-Chaves, Ferdinand Spurny, Tanja Laske, Mhaned Oubounyt, Jan Baumbach

**Affiliations:** 1 Institute for Computational Systems Biology, University of Hamburg, Albert-Einstein-Ring 8-10, 22607 Hamburg, Hamburg Germany; 2 Viral Systems Modeling, Leibniz Institute of Virology, Martinistraße 52, 20251 Hamburg, Hamburg Germany

**Keywords:** Differential networking, Pathway-level differential co-expression, Differential regulation, Disease module identification, Network-based gene expression analysis

## Abstract

Understanding the molecular mechanisms underlying diseases is crucial for more precise, personalized medicine. Pathway-level differential co-expression analysis, a powerful approach for transcriptomics, identifies condition-specific changes in gene-gene interaction networks, offering targeted insights. However, a key challenge is the lack of robust methods and benchmarks specifically for evaluating algorithms’ ability to identify disrupted gene-gene associations across conditions. We introduce *DRaCOoN* (Differential Regulatory and Co-expression Networks), a Python package and web tool for pathway-level differential co-expression analysis. *DRaCOoN* uniquely integrates multiple association and differential metrics, with a novel, computationally efficient permutation test for significance assessment. Crucially, *DRaCOoN* also provides a benchmarking framework for comprehensive method evaluation. Extensive benchmarking on simulated data and three real-world datasets (bone healing, colorectal cancer, and head/neck carcinoma) showed that *DRaCOoN*, particularly with an entropy-based association measure and the *s* differential metric, consistently outperforms eight other methods. It remains highly accurate in balanced datasets, robust to varying gene perturbation levels, and identifies biologically relevant regulatory changes. Furthermore, *DRaCOoN* serves as both a powerful tool and a benchmarking framework for elucidating disease mechanisms from transcriptomics data, advancing precision medicine by uncovering critical gene regulatory alterations.

## Introduction

Understanding molecular mechanisms is fundamental to unraveling biological processes. These processes include disease, developmental stages, healing, and other transitions [1–5]. Changes at the molecular level often drive these processes and, gene expression profiles in particular, offer a rich source of information about cellular activity [6]. Analyzing these profiles illuminates the complex interplay of genes within dynamic biological pathways, which helps in understanding underlying mechanisms and can pinpoint potential therapeutic targets.

Differential network analysis examines how gene interactions change between conditions, for example, healthy versus diseased states [7]. This analysis goes beyond simply identifying genes with altered expression levels. It focuses on the rewiring of gene-gene associations, revealing how relationships between genes are disrupted [2]. TThese changes can include the appearance, disappearance, or inversion of gene-gene interactions. These disrupted interactions may or may not correlate with changes in the expression of individual genes.

Differential co-expression (DC) analysis, a specific type of differential network analysis [8], focuses on changes in gene co-expression patterns across conditions, where co-expression indicates that genes show similar expression patterns, suggesting functional relationships or shared regulatory mechanisms [6]. It reveals dysfunctional sub-networks specific to a disease state and, more generally, how gene interactions rewire in response to perturbations, such as mutations in cancer [9–11]. DC methods can be broadly classified into gene-based, module-based, biclustering, and network-based techniques [8].

Gene-based DC methods quantify how a gene’s associations differ across conditions. Module-based methods analyze changes in co-expression within or between gene modules [8, 12]. These modules are often assumed to be functionally related. For example, if a transcription factor (TF) loses function at the protein level, gene-based methods would identify this TF as differentially co-expressed with its targets [13]. This holds even if the TF’s RNA levels remain stable. Gene-based methods are split into global and local sub-types [14]. Global methods assess a gene’s differential association with all other genes. Local methods evaluate a gene’s differential association with a relevant subset of genes.

Module-based DC analysis methods leverage gene connectivity information, assuming genes cluster within functionally related or co-regulated modules. These modules exhibit higher internal correlation compared to external genes, which reduces data complexity and enhances statistical power. Module-based approaches can identify differential co-expression within or between modules. They are classified by whether modules are predefined or identified from the data and by the number of conditions compared [8]. Biclustering simultaneously clusters genes and conditions to identify condition-specific co-expressed gene modules [8, 12].

Network-based DC analysis methods identify a network of associations that change across conditions. They typically use statistical measures such as correlation or information theory-based measures. Unlike module-based tools that focus on pre-defined or identified groups, network-based methods analyze gene associations individually or across entire networks without such groupings [8].

While module-based and gene-based methods have valuable applications [5, 15], they are less suited for reconstructing complete differential gene regulatory networks. Network-based methods are better suited for this, as they capture the full network and how associations change. Building on network-based methods, differential regulation (DR) analysis adds directionality by focusing on associations between transcription factors (TFs) and their target genes (TGs) across conditions, offering crucial insights into gene regulatory mechanisms underlying diseases [6, 16].

Pathway-level differential co-expression analysis focuses on changes within known biological pathways [17]. Pre-defined pathways can be retrieved from various databases, with a focus on interactions between transcription factors and their target genes. This approach isolates pathway-specific interactions, which is crucial for understanding complex regulatory relationships. Pathway-level differential co-expression analysis examines co-expression changes within gene pairs of specific regulatory pathways across conditions [18]. This enables the construction of a Differential Gene Regulatory Network (DGRN) [7]. A DGRN provides a detailed view of how molecular interactions within pathways are rewired.

### Related works

The lack of benchmarks for pathway-level differential co-expression analysis has been noted [6, 8]. The absence of a gold standard differential network derived from real biological systems hinders objective evaluation of the effectiveness of different algorithms, making it difficult to perform comparative assessments [14]. While simulated data can help, a comprehensive benchmarking suite needs to account for diverse perturbation types beyond gene knockdowns, biologically relevant parameters, and realistic gene numbers. Moreover, the suite should consider that samples within the same condition should be homogenous when performing differential co-expression analysis.

Existing simulation strategies have limitations. Lareau et al. [19] proposed a method for validating module-based differential co-expression methods. Their approach simulates data by randomly assigning genes to modules and then introducing differential co-expression by rewiring edges within specific modules. However, it does not account for overall network topology and primarily focuses on gene knockdowns. The *dcanr* R package [8] also uses simulations [8]. The simulator generates gene expression data with differential co-expression by modeling a gene regulatory network using a system of ordinary differential equations, incorporating gene knockdowns to perturb the network, and solving for steady-state expression levels. However, *dcanr* limits perturbations to gene knockdowns and while it includes functionality for evaluating the reconstruction of DGRNs, it does not enforce homogeneity within conditions. Within-condition heterogeneity can be addressed by identifying subgroups through techniques like biclustering [20]. DC analysis is then performed between these subgroups. However, this is not considered in the *dcanr* simulation strategy.

To address these limitations, we introduce *DRaCOoN* (Differential Regulatory and Co-expression Networks). *DRaCOoN* is a tool designed to reconstruct DGRNs across two conditions. It is available as an online tool at https://prototypes.cosy.bio/dracoon/ and Python package, and the source code is available at https://github.com/fmdelgado/DRACOONpy. *DRaCOoN* employs well-established association metrics (entropy-based and correlation-based) and novel differential metrics to quantify differences between conditions. We also introduce a new benchmarking strategy that simulates gene expression data following a specific network topology and ensuring homogeneous conditions within each group. This allows for a thorough assessment of *DRaCOoN* and other algorithms, which helps identify the optimal strategy for pathway-level differential co-expression analysis. We further validated *DRaCOoN*’s biological relevance using real-world transcriptomics datasets, revealing key insights into its algorithmic capabilities for capturing network rewiring across conditions.

## Methods

### Algorithmic presentation: DRaCOoN

**Fig. 1:**
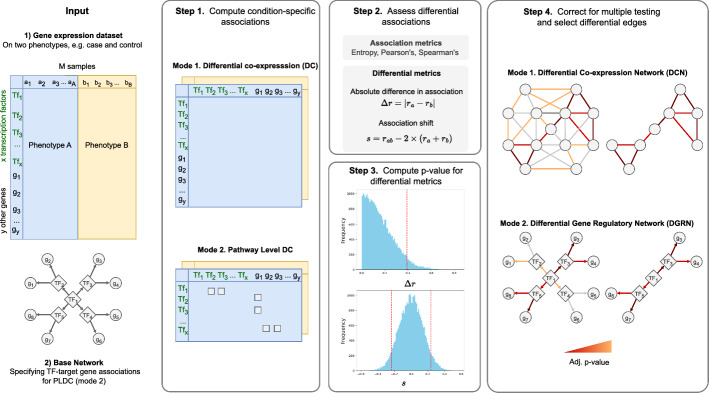
Schematic overview of *DRaCOoN*’s workflow and functionalities. The first step converts an input expression matrix with annotated conditions into a co-expression network represented as a gene-gene matrix. The TF-TG interactions of a GRN, in case of mode 2, are used to construct a DGRN. Thus, we can designate which interactions are evaluated for differential networking: all (mode 1) or TFs-TGs in the GRN (mode 2). In the second step, a background model based on the permutation test is used for estimating the statistical significance of the absolute difference $\Delta r$ and the shift difference *s*. In the third step, we compute the differential metrics for network edges based on a selected association metric (entropy-based, Pearson’s, or Spearman’s) and assign* p*-values to the edges based on the background model. Lastly, in Step 4, the differential edges are corrected for multiple testing. Edges identified as significant constitute the final differential network

*DRaCOoN* is a data-driven method that reconstructs DCNs or DGRNs between two distinct conditions by respectively performing DC or pathway-level DC, as depicted in Fig. 1. In working mode 1, *DRaCOoN* performs DC by examining the association change between conditions for all possible gene-gene associations in the data. The outcome is thus an undirected DC network. In working mode 2 (pathway-level DC), a user-defined subset of transcription factor (TF) - target gene (TG) pairs is used as an additional input for the algorithm. Then the differential examination is only performed on the given TF-TG subset. The outcome is, thus, a directed DGRN. Notably, while *DRaCOoN* in mode 1 is a gene-based global DC approach (see Sect. 1), *DRaCOoN*’s mode 2 can be considered a network-based method that performs pathway-level DC to assess differential regulation by evaluating a set of associations provided by the user.

The input to *DRaCOoN* is an expression dataset obtained through microarray or RNA sequencing (RNA-Seq) technologies, in which multiple samples *M* are screened over one of two conditions *A* or *B*. To ensure that the analysis yields statistically significant and reliable results, it is not only crucial to provide a minimum number of samples per condition but also to ensure these samples are representative and of sufficient quality. This approach helps to mitigate the impact of individual variations, thereby providing results that are not only statistically robust but also relevant and applicable to the broader context of the research question. At least around 10 samples per condition have been suggested by Ballouz et al. [21] and are also recommended for *DRaCOoN*.

*DRaCOoN* estimates differential associations between gene pairs by initially calculating the co-expression of gene pairs under each condition (Fig. 1, step 1). The algorithm allows users to select among Pearson’s correlation coefficient [22], Spearman’s rank correlation coefficient [23], and an entropy metric [24] as measures of co-expression between gene pairs. Comprehensive definitions for these measures can be found in A.1. Following this, the algorithm measures the change in co-expression and computes its statistical significance.

*DRaCOoN* computes two differential metrics, Absolute Difference in Co-expression ($\Delta r$) [25, 26] and Degree of Association Shift (*s*) [24], to provide quantifiable estimates of changes in co-expression values under different conditions (Fig. 1, step 2). These metrics are defined as follows:

**Absolute Difference in Co-expression** ($\Delta r$) The absolute difference $\Delta r$ between the co-expression values under two distinct conditions is calculated using the equation:

1 $$\begin{aligned} \Delta r = \left| r_a - r_b \right| \end{aligned}$$

where the terms $r_a$ and $r_b$ represent the co-expression values under conditions *A* and *B*, respectively. This metric captures the magnitude of change in the association between two genes across the conditions.

**Degree of Association Shift** (*s*) The degree of shift in association between the two conditions is quantified by the metric *s*, calculated as:

2 $$\begin{aligned} s = r_{ab} - 2 \times (r_a + r_b) \end{aligned}$$

where $r_{ab}$ denotes the co-expression value computed for all samples across conditions *A* and *B*.

*DRaCOoN* quantifies the significance of this change using a permutation test-based approach. Traditional permutation-based methods evaluate individual gene pairs by shuffling their values within and between conditions, thus erasing condition-specific differences and calculating *s* and $\Delta r$ using these shuffled values [8, 24, 27]. In our permutation approach, we shuffle the expression values within each condition to retain group-specific differences and disrupt the relationships between genes in each group, followed by the calculation of associations $r_a$ and $r_b$. For the computation of the shift (*s*) groups are also combined and then shuffled for the computation of $r_{ab}$ (see Section A.2 for details). This way of shuffling leads to the loss of group-specific distinctions, and the values obtained from it yield a null model of *s* expected from data without group differences. The process can be repeated $N_p$ times, so we can estimate the* p*-value of the observed differential metric.

Given the high computational demands of the aforementioned method to generate background distributions for all differential edges, we use a single background model for each $\Delta r$ and *s* from which the* p*-values for all evaluated relationships are obtained. This method computes a specific number of permutations, denoted as $N_p$ 10.000 by default, to encompass all differential relationships under study. This way we use the same sets of randomized values as a background model for estimating the significance of differential metrics for all differential edges (Fig. 1, step 2). An edge is considered significant based on the observed $\Delta r$ and *s* values in relation to the corresponding probability density functions of the background models. In the case of $\Delta r$ the test is right-tailed, which means it lies in the extreme right region of the corresponding background model distribution, while in the case of *s* the test is two-tailed, evaluating whether it lies in either the extreme left or right regions of the corresponding background model distribution.

After the differential metrics for all evaluated relationships are assigned their corresponding* p*-value (Fig. 1, Step 3), we correct for multiple testing and apply a final threshold $\alpha$ (0.01 by default) so only significant relationships remain in the analysis (Fig. 1, Step 4). By default, multiple testing correction is done using the Benjamini-Hochberg false discovery rate (FDR-BH) method [28], although users can select any method available in the *statsmodels* Python package [29]. Notably, two differential metrics are obtained for each edge, for $\Delta r$ and *s*, respectively, for which the same *alpha* is applied. Differential relationships can be considered significant according to either or both of these two differential metrics.

The entire process is performed for a set of relationships to evaluate, i.e., all possible gene pairs vs. those within a specific pathway, as selected by modes 1 and 2, respectively. The pseudocode of *DRaCOoN* is provided in Section A.3. The pathway structure to be used in mode 2 can be obtained from several resources, such as ReMap [30], CistromeDB [31], ChIPBase [32], and TRRUST [33], which were conducted to store TF-TG relationships.

## Results

### Benchmarking *DRaCOoN* vs. other methods for pathway-level DC using simulated datasets

**Fig. 2:**
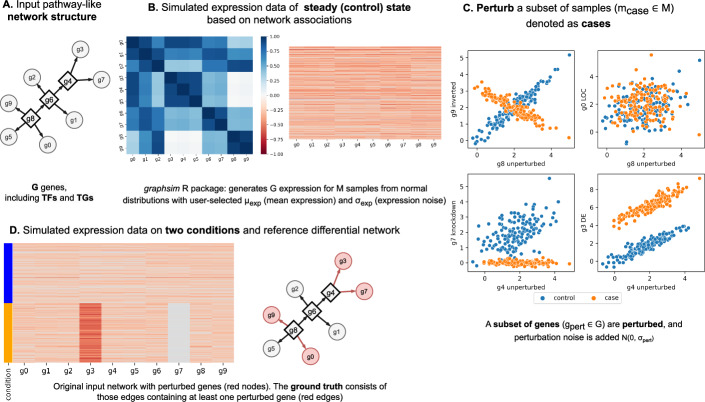
The simulation framework is used for benchmarking. A. The simulation begins initializing a tree-like network structure with user-defined TFs-TGs. In this network, square boxes represent TFs and circles represent target genes. B. The *graphsim* R package simulates gene expression data for a specified number of samples *M* based on network associations, generating a gene-gene similarity matrix and drawing expression values from a multivariate normal distribution. C. The data is split into control and case groups, introducing specific perturbations such as gene knockdown, gene differential expression (DE), inversion, and loss of co-expression (LOC), altering regulatory interactions within the GRN. Additionally, Gaussian noise (N) is added. D. A ground truth network is defined (edges highlighted in red), and details of the perturbed expression data and edges are saved for performance assessment

We simulated data for evaluating the *DRaCOoN* algorithm, which involves generating a random network of TFs and their TGs and simulating gene expression data using the *graphsim* R package [34] based on predefined network structures. This process translates the directed graph into a covariance matrix, reflecting expected correlations between gene expressions. Parameters such as gene expression mean and noise, correlation coefficients, and perturbation types (including gene knockdown, differential expression, inversion, and loss of co-expression) are defined to mimic biological variability and experimental conditions. Perturbations are introduced to the case group samples, simulating various biological disruptions. The simulation generates data for control and case groups, introducing Gaussian noise. This comprehensive approach aims to produce simulated gene expression datasets that closely resemble real biological systems. An overview of our simulation framework is shown in Fig. 2. For a detailed and comprehensive understanding of the simulation framework, see B.1.

Within the simulation framework, we systematically vary certain parameters to explore their impact on the performance of the *DRaCOoN* algorithm in reconstructing DGRNs under different scenarios. We compared *DRaCOoN* vs. other pathway-level DC methods from the *dcanr* R package [8], presented in Table 1 (see B.2 for detailed method description). The parameters varied in the simulations include the ratio of case to control samples and the proportion of genes subjected to perturbations to assess the robustness and adaptability of the *DRaCOoN* algorithm under different simulated biological conditions. To evaluate the performance of each method against the simulation’s ground truth network, we measured effectiveness using the Matthews correlation coefficient (MCC) [35] and identified significant interactions based on a threshold of 0.01 over FDR-BH-corrected* p*-values, a criterion consistently applied across all methods, including *DRaCOoN*-reconstructed networks.

**Table 1:** Overview of methods used for pathway-level DC analysis, adapted from Bhuva et al. [8], all of which have been evaluated together with *DRaCOoN*. These methods have been applied using the authors’ respective implementations found in the *dcanr* R package [8]

Method	Statistical method	Test	Citation
z-score	Correlation	z-test	Zhang et al. [36]
DICER	Correlation	Permutation Test	Amar et al. [27]
DiffCoEx	Correlation	Permutation Test	Tesson et al. [37]
EBcoexpress	Empirical Bayes + correlation	–	Dawson et al. [38]
Entropy	Entropy based on correlation	Permutation test	Ho et al. [24]
GGM-based	GGM + posterior odds	–	Chu et al. [39]
MAGIC	Correlation	Modulation Test	Hsiao et al. [40]
FTGI	Generalised linear model	Chi-squared test	Kayano et al. [41]

**Fig. 3:**
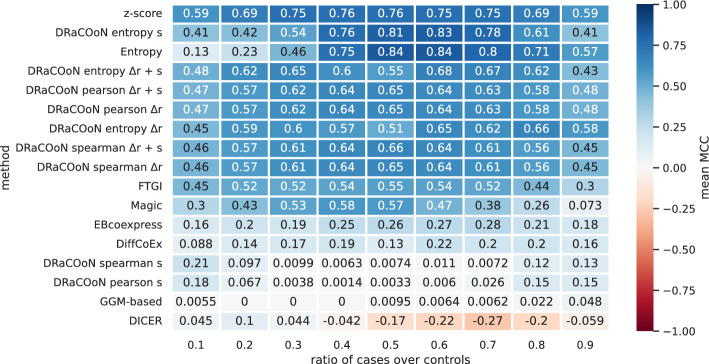
Aggregated MCC for the evaluated pathway-level DC methods, including different *DRaCOoN* configurations, across a spectrum of case-to-total sample ratios. The x-axis represents the ratio of case samples to the total number of samples, ranging from 0.1 to 0.9. Each cell in the heatmap contains the mean MCC score for an algorithm at a specific case-to-total sample ratio, averaged over five runs and five simulations, and the five different perturbation approaches. The algorithms are ranked by their mean performance across all scenarios, and the color gradient reflects the MCC value, with blue intensity indicating higher performance

Figure 3 shows the average method performance in simulations with varied the ratio of case to control samples across a range from 0.1 to 0.9, in steps of 0.1, to examine how different proportions affect the method’s ability to accurately reconstruct DGRNs. As the ratio of cases over the total number of samples becomes closer to 0.5 (i.e., the same proportion of cases and control samples), most network reconstruction methods, like z-score, Entropy, and those employing *DRaCOoN* with the entropy association measure in combination with the *s* differential metric, display an improvement in performance as indicated by rising MCC values. Other methods, such as *DRaCOoN* with the Pearson and Spearman association measures in combination with the $\Delta r$ and the *s* differential metrics, show less variation in MCC values across different case–control ratios and are considered more robust since they are less affected by the proportion of cases and controls, although their general performance is poor. This is also the case for methods like EBcoexpress and DiffCoEx, with poorer performance. Moreover, some methods, like *DRaCOoN* with the Pearson and Spearman association measures in combination with the $\Delta r$ and the *s* differential metrics and DICER, show a decrease in MCC at even proportions of cases and control samples, hinting at loss of generalizability. On the other hand, methods like GGM-based and DICER are consistently at the lower end of the performance spectrum. In Supplementary Figure C2, we display the method’s performance depending on the perturbation approach when varying the ratio of case to control samples.

**Fig. 4:**
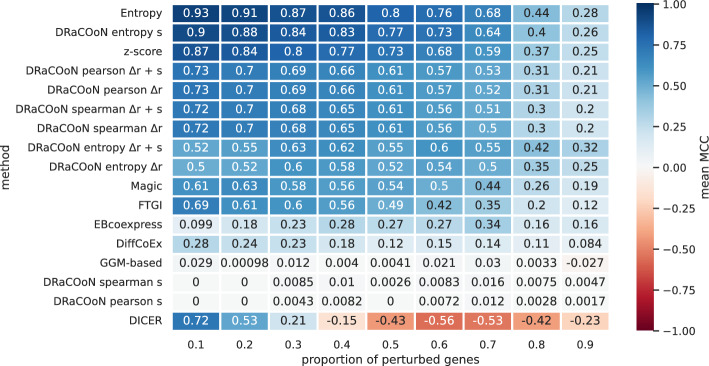
Aggregated MCC the evaluated pathway-level DC methods, including different *DRaCOoN* configurations, with varied proportions of perturbed genes. The x-axis represents the ratio of perturbed genes, ranging from 0.1 to 0.9. Each cell in the heatmap contains the mean MCC score for an algorithm at a specific ratio of perturbed genes, averaged over five runs and five simulations and the five different perturbation approaches. The algorithms are ranked by their mean performance across all scenarios, and the color gradient reflects the MCC value, with blue intensity indicating higher performance

Figure 4 illustrates the impact of varying the proportion of perturbed genes on the performance of different pathway-level DC methods in accurately reconstructing DGRNs. This proportion is varied across a range from 0.1 to 0.9, in increments of 0.1, to explore how an increasing percentage of perturbed genes influences the algorithms’ detection capabilities. As this proportion changes, it challenges the methods to maintain performance in identifying the true altered interactions within the network, providing insight into each algorithm’s sensitivity to the extent of network perturbation. Methods that exhibit minimal variation in MCC scores as the perturbation proportion changes are less sensitive to perturbation levels, indicating potential for more reliable application in diverse practical contexts.

*DRaCOoN* with the entropy association metric, in combination with the *s* differential metric, exhibit very high MCC values (close to 1.0), especially at lower proportions of perturbed genes. This indicates excellent performance, with near-perfect prediction by these methods when perturbations are less frequent. As the proportion of perturbed genes increases, there is a visible gradient of decreasing performance for most methods. However, the decline is not linear; methods like *DRaCOoN* with the entropy association metric in combination with the *s* differential metric and z-score maintain high performance until a specific level of perturbation is reached. The heatmap, in Fig. 4, illustrates that *dcanr*’s Entropy, z-score, and certain configurations of *DRaCOoN*, particularly those utilizing the entropy association metric and the *s* differential metric, maintain robust performance until the proportion of perturbed genes reaches 50%, before declining as the proportion increases. Supplementary Figure C3 showcases the aggregated methods performance as per the various perturbation approaches over the entire range of perturbed gene proportions, reflecting the impact of the perturbation approaches.

### Case study GSE99580: reconstructing time-resolved differential gene regulatory networks upon bone healing

Having established *DRaCOoN*’s performance in simulated data, we next evaluated its ability to identify biologically relevant regulatory changes in a real-world transcriptomics dataset. We utilized the GSE99580 gene expression dataset by Hussein et al. [42], from the Gene Expression Omnibus (GEO) database [43], focusing on bone healing in mice. The original study generated a dataset by inducing closed, stabilized fractures in the femora of 8–12-week-old mice and then providing a timeline of gene expression across various postoperative days (see D.1.1 for further description).

We ran *DraCOoN* for pathway-level DC by leveraging the TRRUST V2 database of TFs-TGs relationships [33], which involved mapping gene identifiers between the dataset and TRRUST V2 using *MyGene.info* [44]. For *DRaCOoN*’s analysis, we utilized 2378 genes from the GSE99580 expression dataset, which were successfully mapped to the TRRUST V2 database, resulting in 6290 regulatory relationships for the pathway-level differential co-expression analysis (see D.1.2 for details).

Using this time-resolved data, we applied *DRaCOoN* to infer DGRNs corresponding to the pairwise comparison for each time point post-operation vs. day 0 as a reference, with the goal of examining the dynamic changes in gene regulation throughout bone healing. As with simulated expression datasets, *DRaCOoN* generated networks using Entropy, Pearson’s, and Spearman’s metrics, selecting significant interactions based on $\Delta r$ and *s* with FDR-BH adjusted* p*-values $< 0.01$, and applying permutation tests for* p*-value calculations. For comparison, we also ran the same *dcanr* methods we ran on simulated data (see D.1.5 for implementation details). Finally, as a reference, we conducted differential expression analysis (DEA), with FDR-BH-corrected* p*-value $< 0.01$, on the 2378 genes from the GSE99580 dataset mapped to TRRUST V2, identifying DEGs for each time point compared to day 0 (D.1.3). Using these DEGs, we reconstructed networks from the 6290 mapped TRRUST V2 interactions by including edges with at least one DEG, assigning the lowest FDR-BH-corrected* p*-value of the involved genes to each edge. These networks are labeled as DEA networks.

For the different time point comparisons, *DRaCOoN* yielded networks of variable sizes (Suppl. Figure D6), depending on the association metric and the differential metric used, which is indicative of dynamic changes in gene interactions over the course of bone fracture healing. Some networks, such as those reconstructed from DEGs and those using entropy as an association metric, consistently show a larger number of edges across all time points, suggesting a more complex interaction model or a less stringent threshold for edge inclusion. Besides, as mentioned in Section 3.2.1, we also performed DEA on the time point comparisons as shown in Supplementary Figures D4 and D5. There is a noticeable trend where the number of DEGs seems to peak at certain time points (e.g., day 0-day 7) and then decreases at later stages (e.g., day 0-day 35), suggesting a dynamic response over time.

#### Node-based over-representation analysis of the GO term for ossification

In order to assess whether the reconstructed DGRNs properly capture the underlying biological processes, we used as a ground truth the *M. musculus* genes associated with the gene ontology (GO) term “ossification” (GO:0001503). From the 109 genes with the GO:0001503, according to the *org.Mm.eg.db* R package [45], only 47 could be identified among the 2378 genes that were mapped between TRRUST V2 and the GSE99580 dataset. We analyzed how well networks identified ossification-related genes at each time point by estimating the overlap between genes comprised within significant edges (over the initial pool of 2378 genes) and the genes associated with ossification. For each time point, we used Fisher’s exact test to determine the significance of the association between network genes and ossification-related genes at each contingency table, using as a cutoff for significance the FDR-BH* p*-value $< 0.01$.

**Fig. 5:**
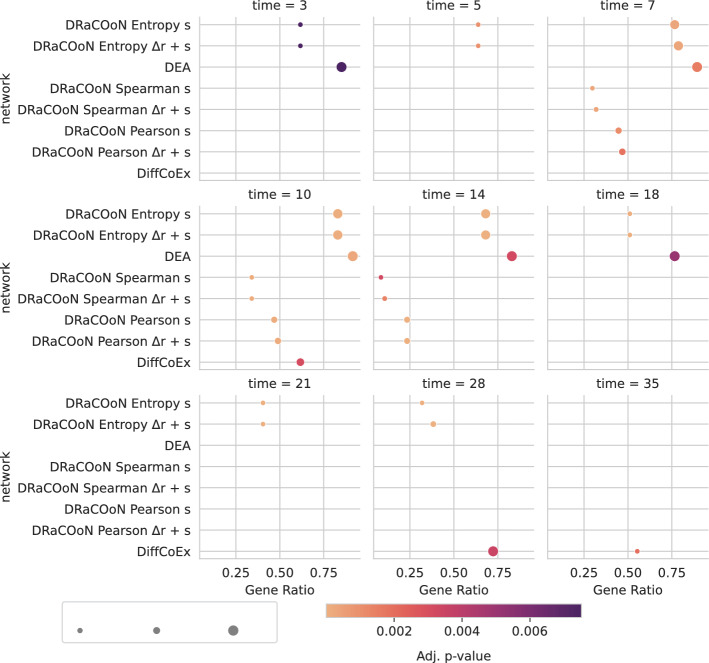
Time-resolved ORA depicting the significance of the reconstructed networks highlighting enrichment on the GO term “ossification” (GO:0001503). Each point corresponds to a gene set captured by each network or DEGs (in the y-axis), and the x-axis shows the gene ratio. The color intensity indicates the Adj.* p*-value from Fisher’s Exact Test, with a gradient color map ranging from low (light) to high (dark) significance, as normalized by the color bar at the bottom. Point size represents the number of genes that represent the GO term, while gene ratio represents the fraction of identified GO-term-related genes over the total number of known GO-term-related genes. Networks at each time point are arranged in a grid, allowing for a comparative view of regulatory changes over time. Only significant interactions (FDR-BH-corrected* p*-value $< 0.01$) are represented

As shown in Fig. 5, ORA revealed distinct trends and patterns of significance in ossification enrichment across different time points and network reconstruction methods. Notably, at days 7 and 10 post-injury, there is a marked peak in significant ossification enrichment, as evidenced by a greater number of statistically significant interactions across most reconstructed networks for that day. Supplementary Figure D7 illustrates a DGRN for day 10. Comparing various network analysis methods, those networks using entropy as the association metric in conjunction with *s* or $s + \Delta r$ as differential metrics consistently yielded significant associations with ossification across all time points, except days 3 and 35 post-surgery. Notably, the most extreme Adj.* p*-values, indicative of the highest statistical significance, were predominantly observed in networks that utilized entropy as the association metric paired with *s* or $s + \Delta r$ as differential metrics, suggesting this metric combination is highly effective in accurately identifying gene regulatory changes associated with ossification. Furthermore, the $\Delta r$ sub-networks exhibited poorer performance compared to their corresponding *s* counterparts.

This analysis shows that entropy-based approaches are more effective than other methods for capturing the regulatory relationships underlying the ossification process. The entropy-based association metric’s effectiveness is attributed to its capacity to uncover complex, non-linear associations that linear or rank correlations may overlook. Crucially, the synergy between the entropy association metric and the *s* differential metric was recommended by [24]. This synergy is likely due to the dataset’s pathway-level differential co-expression being predominantly driven by differential expression patterns, which are more accurately captured through the *s* metric (as simulated in Figure B1). This distinction clarifies that the performance differences observed are primarily due to the differential metrics’ capacity to reflect changes in gene co-expression, highlighting that entropy-based approaches, especially when paired with the *s* differential metric, are particularly effective in delineating the regulatory dynamics underlying the ossification process, especially when driven by differential expression. Figure D8 is an extended version of Fig. 5, including additional bone healing-related GO terms.

#### *DRaCOoN* methods performance in classifying ossification-related edges

Similar to the previous case with simulated data, we evaluated the networks’ ability to retrieve ossification-related edges using MCC. An edge was considered ossification-related if any of its composing genes were also in the ossification gene set. As a comparison, networks were also generated using DEGs. Contingency tables were compiled for each reconstructed network and time point to count edges associated with ossification.

**Fig. 6:**
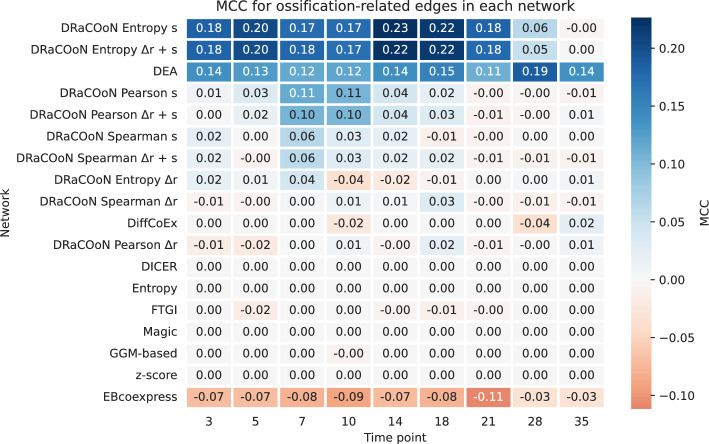
MCC values for classification of ossification-related edges across time points for different networks. Networks are ranked by their mean MCC value to highlight those with the highest predictive performance for bone healing-related interactions. The MCC values are presented on a color gradient ranging from $-1$ to 1, where 1 indicates a perfect prediction, 0 is no better than random chance, and $-1$ indicates an inverse prediction

As shown in Fig. 6, MCC values for each network ranged from $-~0.04$ to 0.23, highlighting the varying abilities of different network reconstruction methods to capture bone healing-related interactions. Notably, networks employing entropy-based measures generally yielded higher MCC values, with the peak performance observed from days 14 to 21 post-operation, which is known to involve several key processes in mice bone healing, including the differentiation of chondrocytes, cartilage extracellular matrix mineralization, recruitment of resorptive osteoclasts, and the initiation of secondary bone formation as the cartilage is resorbed and primary angiogenesis continues to replace the cartilage with nascent bone tissues [46, 47]. In contrast, the networks reconstructed using DEGs demonstrated a more consistent but moderate performance across all time points.

### Case study GSE89076: reconstructing differential gene regulatory networks in colorectal cancer vs. reference tissue

To further demonstrate the utility of *DraCOoN*, we analyzed the GSE89076 gene expression dataset by Satoh et al. [48] from the GEO database [43]. This dataset examines gene expression differences between colorectal cancer and paired normal tissues in patients with colorectal cancer and performed DNA microarray analysis to identify differentially expressed genes (see D.2.1 for further details). The study found that metabolic reprogramming in colorectal cancer is primarily driven by aberrant MYC expression, occurring as early as the adenoma stage.

As in the previous example, we used the TRRUST V2 database of TFs-TGs relationships [33] to run *DraCOoN* for pathway-level DC. This involved mapping gene identifiers between the dataset and TRRUST V2 using *MyGene.info* [44]. We used 2531 genes from the GSE89076 expression dataset for *DRaCOoN*’s analysis. These genes were successfully mapped to the TRRUST V2 database, yielding 7071 regulatory relationships for the pathway-level differential co-expression analysis (for more information, see D.1.2).

In order to investigate the dynamic changes in gene regulation upon colorectal cancer, we used this data to apply *DRaCOoN* to infer DGRNs corresponding to the comparison between tumoral and normal tissue. *DRaCOoN* produced networks using Entropy, Pearson’s, and Spearman’s metrics, as with simulated expression datasets. Significant interactions were chosen based on $\Delta r$ and *s* with FDR-BH adjusted* p*-values $< 0.01$, and permutation test* p*-value computations were used. We also applied the same *dcanr* methods to this real dataset for comparison (for implementation details, see D.2.4). In order to identify DEGs between tumoral and normal tissue, we lastly performed DEA (FDR-BH-corrected* p*-value $< 0.01$), on the 2531 genes from the GSE89076 dataset that were mapped to TRRUST V2 (D.2.3). Similar to Sect. 3.2, by incorporating edges with at least one DEG, we were able to reconstruct networks from the 7071 mapped TRRUST V2 interactions using the results of the DEA (DEA networks). Each edge was then given the lowest FDR-BH-corrected* p*-value of the genes involved.

From the 920 genes with CRC, according to the NeDRex platform [49], which combines data from DisGeNET [50] and OMIM [51], only 290 could be identified among the 2531 genes that were mapped between TRRUST V2 and the GSE89076 dataset. By calculating the overlap between the nodes included in significant edges (over the original pool of 2531 genes) and the CRC-associated genes, we conducted a node-based ORA to measure how well networks identified CRC-related genes in the difference between normal and tumor tissue.

**Fig. 7:**
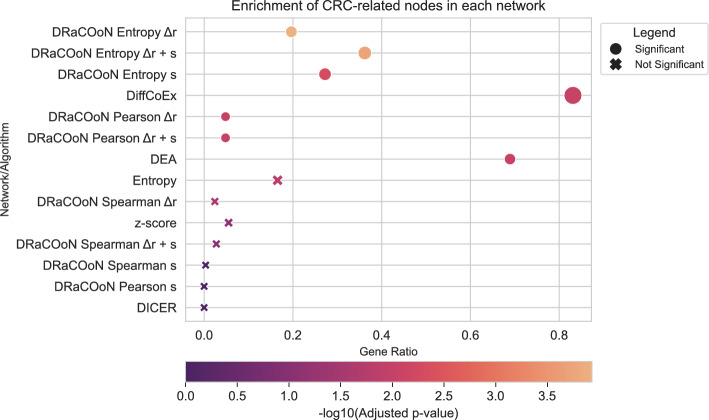
Enrichment analysis of CRC-related nodes across gene networks, visualized using $-\log _{10}$ transformed adjusted* p*-values. The plot displays results from Fisher’s exact tests. Point size reflects the number of overlapping genes. Circles denote significant enrichments (adjusted* p*-value < 0.01), and X markers indicate non-significant results

Fisher’s exact tests were used to determine the statistical significance of the overlap between genes present in each reconstructed network (nodes) and the CRC gene set (Fig. 7). Again, we corrected *p*-values using the FDR-BH method. We also compared these results to those obtained from DEA, where genes were considered significant if their adjusted *p*-value was below 0.01, regardless of their presence in any specific network. Networks reconstructed using DRaCOoN with the entropy association metric consistently outperformed other methods, including DEA, in terms of identifying CRC-related genes. Specifically, the combination of the entropy metric with both the *s* and $\Delta r$ differential metric yielded the most significant enrichment, followed by the entropy metric with the *s* metric alone and then the entropy metric with the $\Delta r$ metric. While all three entropy-based DRaCOoN configurations and DiffCoEx showed significant enrichment (FDR-BH-corrected *p*-value $< 0.01$), the superior performance of the configurations including the *s* metric suggests that differential co-expression in this CRC dataset is, at least in part, driven by shifts in gene association patterns, which are directly quantified by *s*. This finding aligns with previous observations highlighting the efficacy of the *s* metric in pathway-level differential co-expression analyses [24]. A direct comparison highlighting the distinct functional insights gained from the standard DEA versus the DRaCOoN network analysis for this dataset is presented in the Supplementary Material (Section  D.2.6).

### Case study GSE173855: transcriptional subtype shifts in recurrent head and neck squamous cell carcinoma

We extended our analysis to *DraCOoN*’s utility in the comparison between disease states; we analyzed the GSE173855 gene expression dataset by Weber et al. [52] from the GEO database [43]. This dataset investigates gene expression differences between paired primary and relapsed head and neck squamous cell carcinoma (HNSCC) tumors (see Section D.3 for further details). The original study focused on understanding the transcriptional changes associated with HNSCC recurrence and, in particular, the shifts in transcriptional subtypes and their potential implications for treatment. Importantly, the GSE173855 dataset is derived from preprocessed single-cell RNA sequencing (scRNA-seq) data. The authors of the original study performed bulk RNA sequencing on multiple, spatially defined regions and also integrated information from single-cell experiments to classify samples.

As with the previous analyses, we leveraged the TRRUST v2 database of TF-TG relationships [33] as a structural prior for *DRaCOoN*’s pathway-level differential co-expression analysis. Gene identifiers were mapped between the GSE173855 dataset and TRRUST v2 using ‘MyGene.info‘ [44] (see Section D.3.3). This mapping resulted in 2,456 genes from the GSE173855 expression dataset being used for subsequent analyses, corresponding to 7,181 regulatory relationships in the filtered TRRUST v2 network.

To investigate the dynamic changes in gene regulation associated with HNSCC recurrence, we applied *DRaCOoN* to infer DGRNs representing the differences between relapse and primary tumor samples. *DRaCOoN* generated networks using Entropy, Pearson’s, and Spearman’s correlation as association metrics. Significant interactions were selected based on the $\Delta r$ (change in correlation strength) and *s* (shift in correlation) metrics, using an FDR-BH adjusted* p*-value threshold of 0.05, in agreement with the original study’s level of significance. We also used a combined approach. For comparison, we also applied the ‘dcanr‘ methods to this dataset (see Section D.3.5).

We also performed a standard DEA to identify DEGs between relapse and primary tumors, using an FDR-BH adjusted* p*-value threshold of 0.05 (see Section D.3.4). Additionally, we obtained a list of 36 HNSCC-related genes that were present in both the TRRUSTv2 network and the GSE173855 dataset, out of the original 52 from the NeDRex platform [49]. We conducted a node-based ORA to assess the enrichment of these HNSCC-related genes within the networks identified by each method. The ORA calculated the overlap between the nodes (genes) included in significant edges (using the pool of 2,456 mapped genes) and the HNSCC-associated genes. Analogously to Sects. 3.2 and 3.3, and for comparison purposes, we also reconstructed DEA networks by mapping DEGs to the network and giving each edge the lowest FDR-BH-corrected* p*-value of the genes involved.

**Fig. 8:**
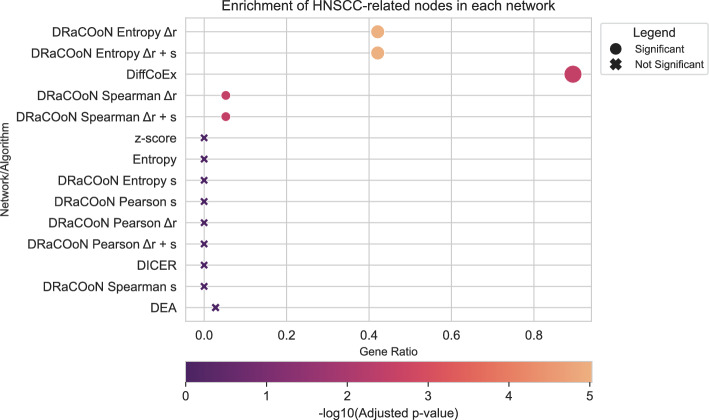
Enrichment analysis of HNSCC-related nodes across gene networks, visualized using -log10 transformed adjusted* p*-values. The plot displays results from Fisher’s exact tests. Point size reflects the number of overlapping genes. Circles denote significant enrichments (adjusted* p*-value < 0.05), and X markers indicate non-significant results

Fisher’s exact tests were used to evaluate the statistical significance of the overlap between the genes in each reconstructed network and the HNSCC gene set (Fig. 8). Resulting* p*-values were corrected for multiple testing using the FDR-BH method. We compared these results to those obtained from the DEA. DRaCOoN networks using the entropy association metric, particularly with the combination of both *s* and $\Delta r$, outperformed the other methods in identifying the relevant genes to HNSCC. As shown in Fig. 8, DRaCOoN networks using the entropy association metric, in particular with the combination of both *s* and $\Delta r$, yielded the most significant enrichment of HNSCC-related genes. The DiffCoEx method also showed statistically significant enrichment. However, while DiffCoEx identified a statistically significant enrichment, the resulting network was considerably larger compared to the DRaCOoN Entropy networks. This suggests that while DiffCoEx captures some relevant genes, it also includes a large number of potentially less relevant genes, making the network less specific to the HNSCC context. DRaCOoN, particularly with the Entropy metric and the combined h *s* and $\Delta r$ approach, provides a more focused and significantly enriched network of HNSCC-relevant genes. The other network methods and the DEA did not perform significant enrichment. For a direct comparison illustrating the functional differences captured by DEA versus the DRaCOoN network approach in this HNSCC context, see Supplementary Material (Section D.3.6).

## Discussion

In our exploration of pathway-level DC methods, the Entropy association metric, often combined with the *s* differential metric, and sometimes with both *s* and $\Delta r$, demonstrated robust performance in simulated data, maintaining high accuracy across balanced datasets and varying levels of gene perturbation. These findings were consistently observed in applications to real-world datasets, including bone healing (GSE99580), colorectal cancer (GSE89076), and head and neck squamous cell carcinoma (GSE173855), aligning with known biological processes and providing valuable insights into disease mechanisms.

### Simulated data

Overall, the highest performance is observed in balanced datasets, with methods like *dcanr*’s Entropy, *DRaCOoN* with the Entropy-based association metric combined with the *s* differential metric, and z-score (Fig. 3, Supplementary Figure C2). Our analysis indicates that detecting pathway-level DC in heavily imbalanced datasets is challenging, likely due to the limited size of one of the groups, underscoring the complexity of network reconstruction under such conditions. The data imbalance challenge is particularly relevant for correlation-based methods like DICER and *DRaCOoN* with Pearson and Spearman association metrics. As the imbalance becomes more extreme, most methods show a decline in performance. This is because uneven representation of cases and controls leads to a biased background model in permutation test-based methods (e.g., DICER, DiffCoEx, Entropy, MAGIC, and all *DRaCOoN* variants). Specifically, when one condition has significantly fewer samples, the number of possible unique permutations of the data is restricted. This limited permutation space can result in an inaccurate null distribution, artificially inflating or deflating the significance of observed correlation changes. Small group sizes reduce statistical power and increase variability, affecting test result reliability [53]. Nevertheless, *dcanr*’s z-score and Entropy, and *DRaCOoN* (with the entropy association metric and the *s* differential metric) achieve their highest performance in balanced datasets.

Most methods exhibit decreased performance as the proportion of perturbed genes increases, particularly beyond 50% (Fig. 4, Supplementary Figure C3). This decline is likely due to the increased complexity of the perturbed network, with more indirect effects and potential spurious correlations. This trend is observed across all tested methods, highlighting a common challenge in differential network analysis. However, *dcanr*’s Entropy and DRaCOoN, especially when using the entropy-based association metric and the *s* differential metric, show greater robustness at lower perturbation levels, maintaining relatively high performance up to a 50% perturbation proportion (Fig. 4). This may be attributed to entropy’s ability to distinguish relevant patterns from random fluctuations in gene expression data, a property that becomes increasingly valuable with larger perturbations [24]. The entropy-based measure is particularly well-suited to capturing non-linear relationships between gene expression levels. Traditional correlation measures, such as Pearson’s and Spearman’s, are primarily sensitive to linear or monotonic relationships. In contrast, the entropy-based measure quantifies the overall “randomness” of the joint expression distribution of two genes. A change in the shape of this joint distribution, even if the linear correlation remains similar, can indicate a change in the regulatory relationship (see Figure B1, lower-right panel). DRaCOoN’s permutation testing approach allows us to assess the significance of these changes. While this highlights a potential limitation for real-life datasets with unknown, potentially large perturbations, our simulation framework, by varying the perturbation proportion, allows us to evaluate each method’s robustness, crucial for understanding their limitations and strengths in real-world applications.

The general decline in performance with increasing perturbation levels raises important considerations for applicability. In practice, the scale of transcriptomic alterations is often extensive and unknown, as seen in the stark contrasts between tumor and non-tumor tissues [54]. Such deviations underscore the complexities in constructing accurate background models for network analysis. Methods like *DRaCOoN*, with the entropy association metric and the *s* differential metric, and *dcanr*’s Entropy [8, 24] are effective and robust across varying perturbation levels.

Our simulation findings emphasize the importance of choosing methods based on sample balance and expected perturbation levels. They also highlight the need for robust methods, especially when applied to heterogeneous groups, common in complex diseases. Advancing network reconstruction methods to be more resilient and reliable is essential for accurate insights into biological networks.

While the z-score method shows good general performance, its ability to infer causation from associations is limited [8]. Z-scores can identify statistical anomalies but may not always capture the biological nuances of differential expression (Figures C2, C3). However, changes in a gene’s expression often have cascading effects, creating multiple differential co-expression effects, even if the gene’s expression change has only a single pairwise interaction or influences the phenotype alone [19]. Therefore, differential expression plays a key role in DC analysis.

### Real-World Data Applications

#### Bone Healing (GSE99580)

Our study leverages *DRaCOoN* to investigate the gene regulatory dynamics of bone healing in mice, utilizing the GSE99580 dataset [42]. Focusing on ossification, we constructed networks across various time points. Networks using the Entropy association metric combined with the *s* differential metric provided unique insights into the temporal dynamics of gene regulation, as evidenced by significant ossification enrichment from days 5 to 21 post-fracture. These networks also showed higher enrichment in ossification-related genes than other methods/networks at the key 7- and 10-day timepoints.

This finding corroborates previous studies [55], identifying core biological processes that undergo temporal changes during fracture healing. The transition from soft to hard callus, starting around day 7, and the completion of callus bridging by day 21 [42], align with our observed enrichment patterns. This also aligns with known phases of bone healing, where the initial inflammatory phase is followed by bone formation and ossification [46, 56].

We acknowledge that the sample sizes at some time points in the GSE99580 dataset are relatively small (less than 10), which may reduce the statistical power to detect all differential co-expression relationships at those specific time points. However, the consistency of the ossification enrichment across multiple time points, including those with larger sample sizes, strengthens the overall biological interpretation and provides valuable insights into DRaCOoN’s performance in a dataset that really reflects a real-world setting. The higher enrichment values for “ossification” using the entropy association metric and the *s* differential metric (Fig. 5) reflect the dynamic cellular and molecular events in bone regeneration. While our main analysis focuses on ossification, a key process in bone healing, the reconstructed networks are also enriched in other GO terms related to additional bone healing processes, as detailed in Salhotra et al. [57] (Supplementary Figure D8). The increased overrepresentation of ossification-specific nodes during intermediate phases (days 7–18) underscores a pivotal stage in bone repair. The fact that ossification is expected to be highly active during bone healing provides a strong argument for its substantial representation in the reconstructed networks.

#### Colorectal Cancer (GSE89076)

The analysis of the colorectal cancer dataset (GSE89076) further validated the effectiveness of *DRaCOoN*, particularly when using the Entropy association metric. The combination of Entropy with both the *s* and $\Delta r$ differential metrics yielded the most significant enrichment of CRC-related genes (Fig. 7). This superior performance suggests that differential co-expression in this CRC dataset is driven by both shifts in gene association patterns (*s*) and changes in the strength of associations ($\Delta r$). This reinforces the utility of the *s* metric, aligning with previous findings [24]. While DiffCoEx also showed significant enrichment, *DRaCOoN*’s Entropy-based methods consistently outperformed it, indicating a higher specificity for CRC-related genes.

#### Head and Neck Squamous Cell Carcinoma (GSE173855)

The HNSCC case study (GSE173855) provided a different biological context, comparing relapse and primary tumors. Again, *DRaCOoN* networks using the Entropy association metric, especially the combination of *s* and $\Delta r$, showed the most significant enrichment of HNSCC-related genes (Fig. 8). While DiffCoEx also exhibited significant enrichment, its resulting network was considerably larger, suggesting a lower specificity compared to *DRaCOoN*. This reinforces the finding that *DRaCOoN*, particularly with the Entropy metric and combined differential metrics, can provide a more focused and relevant network, even in the absence of a strong temporal component like that present in the bone healing dataset.

#### Overall Trends and Comparisons

Across all three real-world datasets, DRaCOoN, particularly with the Entropy association metric, consistently demonstrated strong performance in identifying relevant gene sets. Often the best-performing configuration included the *s* differential metric (alone or in combination with $\Delta r$). This consistent performance across diverse biological contexts (bone healing, CRC progression, HNSCC recurrence) highlights the robustness and general applicability of this approach. The strong performance of configurations including the *s* metric, especially when combined with Entropy, suggests that shifts in gene association patterns are a crucial aspect of differential co-expression in these biological processes. This supports the idea that changes in gene expression levels (captured by *s*) often lead to broader changes in regulatory interactions.

The comparison with other methods, particularly DiffCoEx, consistently showed that while other methods can identify relevant genes, DRaCOoN (with Entropy) often provides a more focused and significantly enriched network (Figures D11, D15). The larger network sizes produced by methods like DiffCoEx suggest a trade-off between sensitivity (capturing more potential interactions) and specificity (focusing on the most relevant interactions). This difference in focus between methods extends beyond network structure to the functional insights derived. It is crucial to recognize that standard DEA and differential network analysis provide complementary, rather than directly comparable, perspectives. As shown in our analyses for the CRC and HNSCC datasets (Supplementary Sections D.2.6 and D.3.6, respectively), DEA effectively identifies broad cellular processes heavily impacted by disease (e.g., proliferation, metabolism) by focusing on individual gene expression changes. Conversely, DRaCOoN’s network-based approach, which inherently assesses alterations in gene-gene interactions, tends to yield stronger enrichment for specific regulatory, transcriptional, and developmental pathways (e.g., Figures D12 and D16). This interaction-centric view, rooted in the analysis of the network itself, offers potentially deeper mechanistic insights into the underlying dysregulation, complementing the feature-level view provided by DEA.

Our analysis demonstrates that the choice of method is crucial for inferring reliable differential GRNs. The entropy association metric appeared to be particularly effective at identifying meaningful patterns. Additionally, when performing pathway-level DC analysis, it is desirable to use a differential metric that also captures patterns due to differential expression. This can be achieved using the differential metric *s*, ensuring a more comprehensive assessment of the regulatory relationships compared to $\Delta r$ alone [6, 19]. Differential gene expression can lead to changes in its interactions with other genes, resulting in differential co-expression patterns [8]. Notably, the entropy association metric, when paired with the *s* differential metrics, often yields considerably larger networks in both nodes and edges (Figure D6), which may be attributed to many differential edges caused by differential expression changes. When incorporating the *s* differential metric, pathway-level DC analysis can also identify DC edges due to differential expression, whereas the latter alone focuses on the individual affected genes [58]. Furthermore, differential gene expression can affect the formation of co-expression modules or networks, leading to the identification of gene sets with condition-specific regulatory relationships. These modules may represent functional units that respond differently under distinct conditions, e.g. an environmental stimulus, which can be observed using pathway-level DC [9].

It is important to acknowledge several limitations inherent in our approach, particularly concerning the gene mapping process and the completeness of the reference regulatory network. Our analysis relies on the TRRUST v2 database [33], a valuable and widely-used resource of curated transcriptional regulatory interactions. However, like all such databases, it represents a subset of known regulatory relationships, focusing primarily on well-characterized genes and interactions. Consequently, the reconstructed differential networks presented here are inherently dependent on the structure and content of TRRUST v2. Genes present in the original expression datasets (GSE99580, GSE89076, and GSE173855) that were not represented in the filtered TRRUST v2 network, either due to a lack of known regulatory interactions or due to challenges in identifier mapping, could not be included in the network reconstruction. This unavoidable data loss, a long-standing issue in the field, has an impact on the representativeness of the reconstructed networks because it limits our ability to capture the full complexity of the underlying biological processes. While we employed established tools (MyGene.info) to maximize mapping efficiency, a proportion of genes inevitably remained unmapped. Results are best understood as representations of differential co-expression within the *characterized* portion of the regulatory network, rather than a complete picture of all possible gene-gene interactions. Future advancements in regulatory network mapping and more comprehensive databases will be crucial for addressing this limitation and improving the accuracy and completeness of pathway-level differential co-expression analyses.

### Future directions and conclusion

While *DRaCOoN* has shown promising results in the context of bulk transcriptomics, its design holds the potential for broader applications across different quantitative omics technologies. Currently, its validation has been confined to gene-level transcriptomics data. However, the principle underlying *DRaCOoN* is applicable to similar datasets from other omics fields, such as proteomics, alternative splicing of RNA, or single-cell RNA sequencing. Recognizing the versatility and adaptability of *DRaCOoN*, future work will aim to extend its application to these diverse datasets. This expansion will not only enhance the utility of *DRaCOoN* but also contribute to a more comprehensive understanding of complex biological processes through the lens of various omics technologies.

In conclusion, *DRaCOoN* represents a significant advancement in pathway-level differential co-expression analysis, with configurations using the Entropy association metric and the *s* differential metric (alone or combined with $\Delta r$) offering robust and insightful analyses across both simulated and real datasets. Its innovative approach to* p*-value estimation allows for a methodologically efficient and accurate exploration of gene regulatory networks, highlighting important dataset features such as class balance and gene perturbation levels. *DRaCOoN* highlights the significance of differential network analysis in clarifying the intricate interplay of gene interactions between different conditions and establishes the framework for its application in the elucidation of disease mechanisms and the mechanistic understanding of intricate biological processes. This opens the door to focused therapeutic approaches and a deeper comprehension of the molecular basis of diverse disorders.

## Supplementary Information

Supplementary file 1

## Data Availability

The algorithms and analytical methods used in this study are encapsulated within the *DRaCOoN* software package, which is openly accessible under an open-source license. The source code, comprehensive documentation, and illustrative examples are available in our GitHub repository at https://github.com/fmdelgado/DRACOONpy. Furthermore, the *DRaCOoN* package is also distributed through the Python Package Index (PyPI) https://pypi.org/project/dracoonpy/ and can be easily installed with the command pip install dracoonpy. In addition, we offer a web-based interface for the *DRaCOoN* tool, designed to facilitate researchers without a computational background to conduct network reconstruction analyses. This web tool is accessible at https://prototypes.cosy.bio/dracoon/ and provides an interactive environment for analysis execution. All materials used in this study are commonly available.
