# Supplementary material for: DRaCOon: a novel algorithm for pathway-level differential co-expression analysis in transcriptomics
Source: BMC Bioinformatics. 2025 May 26;26:137. doi: 10.1186/s12859-025-06162-9 (PMC12107744; doi:10.1186/s12859-025-06162-9)
Supplement: Supplementary file 1 [file 12859_2025_6162_MOESM1_ESM.pdf]

Supplementary Methods

A. Algorithm implementation details

A.1. Co-expression Metrics Included in *DRaCOoN*

The correlation between genes is a critical aspect of genomic studies. *DRaCOoN* offers three robust metrics to measure gene co-expression: Pearson, Spearman, and entropy association metrics. Each metric offers different insights into various types of gene relationships.

Pearson's Correlation Coefficient (r)

This metric quantifies the degree of linear correlation between two variables, X and Y. It is defined mathematically as:

$$r_{X,Y} = \frac{\text{cov}(X,Y)}{\sigma_X \sigma_Y} \tag{A1}$$

Here X and Y are vectors of observations, i.e., gene expression values across samples. The symbols σ_X and σ_Y denote the standard deviations of X and Y, respectively, while $\text{cov}(X,Y)$ represents their covariance. Pearson's coefficient ranges from -1 (perfect negative linear correlation) to +1 (perfect positive linear correlation), with 0 indicating no linear correlation.

Spearman's Rank Correlation Coefficient (ρ)

Spearman's coefficient, ρ, assesses monotonic relationships between two variables X and Y. Unlike Pearson's, which focuses on linear relationships, Spearman's method ranks the variables and calculates the Pearson correlation of these ranks. The formula is as follows:

$$\rho_{X,Y} = \frac{\text{cov}(R(X), R(Y))}{\sigma_{R(X)} \sigma_{R(Y)}} \tag{A2}$$

In this equation, $R(X)$ and $R(Y)$ are the rank values of X and Y, with $\text{cov}(R(X), R(Y))$ being their covariance. The standard deviations of these ranks are represented by $\sigma_{R(X)}$ and $\sigma_{R(Y)}$. A Spearman coefficient of 1 or -1 indicates a perfect monotonic relationship, whereas 0 suggests no such relationship.

Entropy-Based Correlation (r_{ent})

Entropy, in this context, evaluates the uncertainty or randomness in the relationship between two variables. If one variable is predictable based on another, the system has low entropy, indicating a stronger relationship. High entropy suggests a lack of association. This metric is particularly useful when the relationship is non-linear, where traditional correlation coefficients like Pearson's may fall short. The entropy-based measure in *DRaCOoN*, derived from the Pearson correlation r, is calculated as:

$$r_{\text{ent}} = -\left[\frac{1+|r|}{2}\log_2\left(\frac{1+|r|}{2}\right) + \frac{1-|r|}{2}\log_2\left(\frac{1-|r|}{2}\right)\right] \tag{A3}$$

This formula offers a nuanced view of gene co-expression by incorporating the magnitude of the Pearson correlation r into an entropy framework, providing an alternative perspective on gene relationships.

A.2. Permutation Testing and P-value Estimation

DRaCOoN employs a novel permutation testing approach to assess the statistical significance of the observed differential co-expression metrics (Δr and s). DRaCOoN shuffles expression values within and across conditions. This approach preserves condition-specific characteristics while disrupting gene-gene relationships.

Specifically, for each gene pair (let's denote them as genes X and Y), we perform the following steps:

1. **Within-condition shuffling:** Expression values for each gene (X and Y) are randomly shuffled within each condition (A and B separately). Let X_a and Y_a represent the expression values of genes X and Y in condition A, and X_b and Y_b represent the expression values in condition B. The shuffling operation can be represented as:

$$X_a^{'} = \text{Shuffle}(X_a), \quad X_b^{'} = \text{Shuffle}(X_b), \quad Y_a^{'} = \text{Shuffle}(Y_a), \quad Y_b^{'} = \text{Shuffle}(Y_b) \tag{A4}$$

   where Shuffle() denotes a random permutation of the input vector. This maintains the overall distribution of expression levels within each condition but breaks any existing co-expression patterns.
2. **Calculation of condition-specific co-expression (r_a' and r_b'):** After within-condition shuffling, we calculate the co-expression between the shuffled gene expression values for each condition using the chosen association metric (AM, which can be Pearson's, Spearman's, or the entropy-based measure):

$$r_a^{'} = \text{AM}(X_a^{'}, Y_a^{'}), \quad r_b^{'} = \text{AM}(X_b^{'}, Y_b^{'}) \tag{A5}$$

3. **Across-condition shuffling for s:** To calculate the null distribution for the s statistic, we perform an additional shuffling step after combining the already shuffled samples from both conditions. This creates a combined dataset where condition labels are effectively randomized. We concatenate the shuffled expression values from both conditions:

$$X^{'} = \text{Shuffle}(X_a^{'}|X_b^{'}), \quad Y^{'} = \text{Shuffle}(Y_a^{'}|Y_b^{'}) \tag{A6}$$

   where | denotes concatenation of the vectors.
4. **Calculation of combined co-expression (r_{ab}'):** We then calculate the co-expression between the genes using the combined, shuffled data:

$$r_{ab}^{'} = \text{AM}(X^{'}, Y^{'}) \tag{A7}$$

5. **Calculation of permuted $\Delta r'$ and s':** Finally, we calculate the permuted differential co-expression metrics:

$$\Delta r^{'} = \left| r_a^{'} - r_b^{'} \right|, \quad s^{'} = \frac{r_a^{'} + r_b^{'}}{2} - r_{ab}^{'} \tag{A8}$$

6. **Iteration:** Steps 1–5 are repeated N_p times (default: 10,000) to generate a null distribution for both Δr and s.

To ensure computational efficiency, particularly when analyzing large datasets, DRaCOoN utilizes two background models, one for each differential metric (Δr and s). These background models are generated by performing N_p permutations and obtaining values respectively for Δr and s using randomly selected pairs of genes from the entire dataset. This approach is justified because the null distribution of Δr and s is primarily determined by the overall distribution of expression values, the sample sizes, and the chosen association metric, not the specific gene pair being considered. By using comprehensive background models, we avoid the computational burden of generating separate null distributions for each gene pair while still maintaining the statistical validity of the permutation test. The p-value for each observed Δr and s is then estimated by comparing the observed value to the corresponding background distribution. For Δr, a right-tailed test is used (only values greater than the observed value are counted), while for s, a two-tailed test is employed (values both greater and smaller than the observed value are counted).

A.3. *DRaCOoN* Algorithm Pseudocode

This section provides a simplified pseudocode representation of the *DRaCOoN* algorithm. *DRaCOoN* is designed to identify differential co-expression or differential regulatory relationships between two conditions, such as case and control, in gene expression data.

Algorithm 1 *DRaCOoN* Requirements

Require:

expression_data (a matrix with samples in rows and genes/biomolecules in columns)
condition_data (a data frame indicating the condition of each sample)
program_mode ∈ {'DC', 'DR'} (DC: Differential Co-expression, DR: Differential Regulation)
association_metric ∈ {'pearson', 'spearman', 'entropy'}
significance_level (α)
pval_method ∈ {'permutation', 'background'}
pval_adj_method (e.g., 'fdr_bh' for False Discovery Rate Benjamini-Hochberg)
TF_TG_network (a data frame with 'source' and 'target' columns defining TF-TG interactions, required if program_mode = 'DR')
iters (number of iterations for 'permutation' or 'background' methods)

Algorithm 2 *DRaCOoN* Main Algorithm

```
Ensure: differential_network (a data frame representing the significant differential network)
Preprocessing:
conditions ← unique values in condition_data['condition']   ▷ Get the unique condition labels
data_A ← subset of expression_data where condition = conditions[0]   ▷ Subset data for the first condition
data_B ← subset of expression_data where condition = conditions[1]   ▷ Subset data for the second condition
if program_mode = 'DR' then
    source_targets ← (source, target) pairs from TF_TG_network present in expression_data   ▷ Use predefined TF-TG pairs
else
    source_targets ← all unique pairs of genes in expression_data   ▷ Consider all possible gene pairs
end if
if pval_method = 'background' then
Estimate Background Model:
    background_absdiff, background_shift ← GenerateBackground(data_A, data_B, association_metric, iters)   ▷ Generate background distributions for p-value calculation
end if
Calculate Differential Associations:
results_table ← empty table
for all (source, target) in source_targets do
    r_A, r_B, r_all ← CalculateAssociation(data_A, data_B, expression_data, source, target, association_metric)   ▷ Calculate associations in each condition and overall
    absdiff ← |r_A - r_B|   ▷ Calculate the absolute difference in association between conditions
    shift ← (r_A + r_B) / 2 - r_all   ▷ Calculate the shift in association
    if pval_method = 'permutation' then
        p_absdiff, p_shift ← PermutationTest(data_A, data_B, source, target, association_metric, iters)   ▷ Calculate p-values using permutation test
    else if pval_method = 'background' then
        p_absdiff ← TestSignificance(absdiff, background_absdiff, False)   ▷ Calculate p-value for absdiff using background distribution (one-sided)
        p_shift ← TestSignificance(shift, background_shift, True)   ▷ Calculate p-value for shift using background distribution (two-sided)
    end if
    Add (source, target, r_A, r_B, r_all, absdiff, p_absdiff, shift, p_shift) to results_table   ▷ Store results for this gene pair
end for
Postprocessing:
differential_network ← results_table
Add adjusted p-values (padj_absdiff, padj_shift) to differential_network using pval_adj_method on (p_absdiff, p_shift)   ▷ Correct p-values for multiple testing
Filter differential_network: keep rows where padj_absdiff < α or padj_shift < α   ▷ Keep only significant interactions
Return differential_network
```

Algorithm 3 DRaCOoN Auxiliary Procedures I

```
procedure GenerateBackground(data_A, data_B, association_metric, iters)
    background_absdiff ← [], background_shift ← []    ▷ Initialize empty lists for background distributions
    for i = 1 to iters do
        gene_i, gene_j ← randomly select two genes from data_A or data_B
        sim_abs_dif, sim_shift ← SimulateValues(data_A, data_B, gene_i, gene_j, association_metric)    ▷ Get simulated values
        Append sim_abs_dif to background_absdiff, sim_shift to background_shift
    end for
    return sorted(background_absdiff), sorted(background_shift)    ▷ Return sorted background distributions
end procedure

procedure SimulateValues(data_A, data_B, gene_i, gene_j, association_metric)
    values_A_i, values_B_i ← data_A[gene_i], data_B[gene_i]    ▷ Get expression values for gene_i in both conditions
    values_A_j, values_B_j ← data_A[gene_j], data_B[gene_j]    ▷ Get expression values for gene_j in both conditions
    shuff_values_A_i, shuff_values_B_i ← Permute(values_A_i), Permute(values_B_i)    ▷ Permute the values within each condition
    shuff_values_A_j, shuff_values_B_j ← Permute(values_A_j), Permute(values_B_j)    ▷ Permute the values within each condition
    sim_r_A ← CalculateAssociation(shuff_values_A_i, shuff_values_A_j, association_metric)    ▷ Calculate association for condition A with permuted data
    sim_r_B ← CalculateAssociation(shuff_values_B_i, shuff_values_B_j, association_metric)    ▷ Calculate association for condition B with permuted data
    combined_i ← Concatenate(shuff_values_A_i, shuff_values_B_i)    ▷ Combine permuted values for gene_i
    combined_j ← Concatenate(shuff_values_A_j, shuff_values_B_j)    ▷ Combine permuted values for gene_j
    sim_r_all ← CalculateAssociation(combined_i, combined_j, association_metric)    ▷ Calculate association for the combined/permuted data
    return |sim_r_A - sim_r_B|, (sim_r_A + sim_r_B) / 2 - sim_r_all    ▷ Return simulated absdiff and shift
end procedure
```

Algorithm 4 DRaCOoN Auxiliary Procedures II

```
procedure CalculateAssociation(data_A, data_B, expression_data, source, target, association_metric)
    if association_metric = 'pearson' then
        r_A ← PearsonCorrelation(data_A[source], data_A[target])
        r_B ← PearsonCorrelation(data_B[source], data_B[target])
        r_all ← PearsonCorrelation(expression_data[source], expression_data[target])
    else if association_metric = 'spearman' then
        r_A ← SpearmanCorrelation(data_A[source], data_A[target])
        r_B ← SpearmanCorrelation(data_B[source], data_B[target])
        r_all ← SpearmanCorrelation(expression_data[source], expression_data[target])
    else if association_metric = 'entropy' then
        r_A ← EntropyBasedAssociation(data_A[source], data_A[target])
        r_B ← EntropyBasedAssociation(data_B[source], data_B[target])
        r_all ← EntropyBasedAssociation(expression_data[source], expression_data[target])
    end if
    return r_A, r_B, r_all                ▷ Return the calculated association metrics
end procedure

procedure PermutationTest(data_A, data_B, source, target, association_metric, iters)
    p_absdiff, p_shift are calculated by running iters times SimulateValues(data_A, data_B, source, target, association_metric) and then comparing the simulated distribution to the values of absdiff, shift obtained from the original data.     ▷ Detailed explanation in the pseudocode
end procedure

procedure TestSignificance(observed_value, simulated_results, two_sided)
    if two_sided then
        p_value ← 2 * min(proportion of simulated_results ≥ observed_value, proportion of simulated_results ≤ observed_value)     ▷ Two-tailed test
    else
        p_value ← proportion of simulated_results ≥ observed_value     ▷ One-tailed test
    end if
    return p_value
end procedure
```

B. Simulated data and benchmarking against other methods

B.1. Generation of synthetic data

Since in *DRaCOoN*'s mode 1, no modularity is assessed before DC analysis, genes without modules are cross-correlated. This leads to an increase in noise and potentially a reduction in statistical power. We focus on *DRaCOoN*'s mode 2 only since our goal is to evaluate the ability of *DRaCOoN* against other methods for network-based DC reconstruction. We present a simulation framework specifically created for comparing *DRaCOoN*'s mode 2 to alternative approaches that compute differential associations between gene pairs among different conditions within a given set of network relationships.

B.1.1. Generating gene expression data from a TFs-TGs network

The simulation begins by generating a random network composed of transcription factors (TFs), then adding directed edges from each TF to its target genes, where nodes represent genes and TFs, and edges indicate regulatory relationships, with TFs regulating genes and other TFs, as presented in Figure 2 A. The predefined network structure is inputted into the *graphsim* R package [34], which generates continuous gene expression data for G genes across M samples, ensuring that the expression patterns reflect the relationships and hierarchy defined in the input network (Figure 2 B). To accomplish this, *graphsim* translates the directed graph structure (defined by the network) into a covariance matrix. This matrix represents the expected correlations between gene expressions. The translation considers various parameters to control how the graph structure is interpreted and how it influences the correlations between different genes in the network: The *cor* parameter sets the correlation coefficient to 1, implying a perfect positive correlation. The *dist* parameter considers the physical or topological distance between genes, with more distant genes having lower correlations. The *comm*, *absolute*, and *Laplacian* parameters further refine the analysis by considering community structures, ensuring positive correlations, and using the graph's Laplacian matrix.

The mean μ_{exp} (gene mean expression level) and standard deviation (σ_{exp}, gene expression noise) are used to define the central tendency and the spread of the expression levels for each gene. Using the computed covariance matrix, along with the specified μ_{exp} and σ_{exp} for each gene, the function samples expression levels from a multivariate normal distribution. This process generates a matrix where each row corresponds to a gene and each column to a sample, with values representing the expression levels. The expression levels of genes are correlated according to the network structure and parameters specified, reflecting both the direct relationships in the network and the specified level of noise and average expression. This simulated data mimics real-world gene expression patterns in biological systems, considering both genetic interactions and experimental variability.

B.1.2. Perturbation strategy

Subsequently, we split the simulated expression data into two groups: control and case ($m_{control}, m_{case} \in M$), based on a ratio determined by the user. Following the work by Bhuva et al. [8] and Lareau et al. [19], we apply node-based perturbations to obtain perturbed edges in the gene regulatory network. In those perturbations, we also apply "perturbation noise" (σ_{pert}), a parameter referring to the standard deviation of a normal distribution with a mean of 0, which is used to introduce variability or randomness into the simulated gene expression data, thereby mimicking natural fluctuations observed in biological systems. In our simulation process, we specify the ratio of perturbed genes ($g_{pert} \in G$) over the total number of genes and the type of perturbation. The types of perturbations include gene knockdown, differential expression, inversion of co-expression, and loss of co-expression. Each perturbed gene can only be affected by one of the four perturbation types, which are displayed in Figure 2 C. These perturbations, crucial for mimicking real biological disruptions, are implemented as follows:

1. Gene knockdown: simulates the loss of a gene's expression in the case condition compared to the control. We model this by setting the expression values of affected genes to zero and then adding a random value sampled from a normal distribution with a mean of 0 and a standard deviation equal to 'perturbation noise'.
2. Gene differential expression (DE): this perturbation corresponds to the upregulation of a gene's expression in the case condition relative to the control condition. It is modeled by adding values to the gene's expression profile, resulting in both a directional shift in gene expression and a degree of randomness to more accurately mimic biological variability. In this process, we generate new expression values for genes under perturbation by sampling from a normal distribution. The mean of this distribution is calculated by multiplying the gene's original mean expression level by a factor, denoted as *beta*. The standard deviation of this distribution is set to the value 'perturbation noise'. This approach ensures that the new expression values reflect both the systematic shift induced by the factor *beta* and the randomness introduced by the perturbation noise.
3. Inversion of co-expression pattern: This perturbation method is designed to reverse the co-expression pattern between two genes, effectively changing their correlation from positive to negative, or vice versa. This simulates situations where gene interactions undergo significant changes, such as shifting from synergistic to antagonistic relationships. To achieve this in the simulation, we generate a new vector that is methodically adjusted to be orthogonal to the original gene expression vector. This orthogonal vector is then precisely scaled to achieve a predefined negative correlation, set at -1 with the original vector. Again, random values sampled from a normal distribution with a mean of 0 and a standard deviation equal to 'perturbation noise' are applied to mimic biological variability.
4. Loss of co-expression (LOC) pattern: This perturbation occurs when a consistent co-expression pattern in one condition is absent in another, symbolizing disrupted gene interactions. Loss of co-expression is simulated by shuffling the gene labels of the expression values and adding perturbation noise.

In Figure B1, we directly compare the co-expression metrics above in an example on simulated data, also using the differential metrics $\Delta r\ s$ introduced in Section 2.1 of the main text. As shown, the entropy metric was introduced because it can encompass both correlation-based scores and generalize them to larger sets of genes. Entropy measures the randomness or unpredictability of a variable's outcome, with higher entropy indicating a more unpredictable process and lower entropy indicating a more predictable one [24]. In the context of gene expression, if a set of genes is well correlated, the scatter plots of their expressions will describe narrow ellipsoids, indicating a few large eigenvalues among many smaller ones from the correlation matrix. These eigenvalues can be standardized to sum to one and treated as probabilities. A distribution with one nearly certain outcome (corresponding to the long axis of the scatterplot) will have low entropy. Conversely, if the pooled data from all classes fills a broad ellipse, indicating less correlation, the pooled correlation matrix will yield high entropy. Overall, the entropy-based approach is useful in identifying sets of genes with significant differences in correlation between pooled and class-specific data, using a permutation test for significance assessment. This method shows promise for comprehensive searches of gene sets, particularly when dealing with three-gene combinations, since it accounts for multidimensional relationships that are not captured by simpler correlation-based methods.

B.1.3. Ground truth

In our simulation, the ground truth network is generated to include all edges from the original network structure that involve at least one perturbed gene. For each initialization of the simulation, we conduct five distinct runs. In each of these runs, a subset of genes $g_{pert} \in G$ is selected for perturbation. The first four runs are dedicated to individually exploring each of the four perturbation types. In these runs, all selected genes g_{pert} undergo the same type of perturbation, providing a focused analysis of each perturbation type's impact. In contrast, the fifth run introduces a composite situation where the set of perturbed genes is equally divided among the four perturbation types. This division allows for a simultaneous investigation of all perturbation types within a single run, with each gene g_{pert} being randomly assigned to one of the perturbation types. We generated a variety of datasets and established a set of baseline parameters (Table B1) to guide the creation of our simulated datasets. Starting with the aforementioned baseline parameters, we conducted simulations encompassing two scenarios corresponding to the tuning of two key parameters: the proportion of case and control samples and the proportion of perturbed genes. This means, while the tuned parameter in each scenario can take the range of values in Table B1, the other non-tuned parameters take the baseline value. To comprehensively assess the impact of different perturbations and assessments, each simulated data set is composed of a combination of tuned simulation parameters and one of the 5 possible gene perturbation approaches (4 with a perturbation of each type and 1 with all of them).

B.1.4. Variation of simulation parameters

In our simulation framework, we have set baseline values for several parameters that define the structure and dynamics of the gene regulatory networks. However, to mirror

the variability encountered in real biological datasets and to test the robustness of different network reconstruction methods, we have selectively tuned two parameters: the ratio of case samples ($|m_{case}|/|M|$), and the ratio of perturbed genes ($|g_{pert}|/|G|$), as shown in Table B1.

The ratio of case samples was tuned across a range from 0.1 to 0.9 with a step of 0.1. This tuning is reflective of the varied representation of case conditions in biological datasets, where the proportion of affected individuals can vary widely due to factors such as sample availability and study design. By exploring this range, our simulations can encapsulate situations that challenge the network reconstruction methods to maintain accuracy despite unbalanced data.

The ratio of perturbed genes was similarly tuned from 0.1 to 0.9 with a step of 0.1, which is essential for simulating the unpredictable nature of gene perturbation in biological systems. In actual biological contexts, the proportion of the GRN that is affected by a specific condition or perturbation is generally unknown *a priori*, necessitating a methodological approach that can handle varying degrees of perturbation. This variability is crucial for the robustness of network reconstruction methods, challenging the retrieval of differential gene-gene interactions under diverse and unpredictable conditions.

Finally, for each set of simulation parameters, we generated five simulations in order to account for the inherent variability of the data simulation process. This would result in a total of 450 simulated datasets: length of the range of tuned parameter $\times 5$ possible perturbation approaches $\times 5$ simulations.

Table B1: Baseline parameters in the simulated framework and tuned parameters for simulation scenarios. The "Tuning" column indicates the range of values tested for each parameter, expressed as (Start - Stop, Step).

Parameter	Baseline Value	Tuning
Number of genes ($\|G\|$)	100	-
Ratio of TFs over total number of genes ($\|TFs\|/\|G\|$)	0.3	-
Number of Samples ($\|M\|$)	200	-
Mean gene expression (μ_{exp})	0	-
Expression noise (σ_{exp})	1	-
Perturbation noise (σ_{pert})	1	-
Ratio of case samples ($\|m_{case}\|/\|M\|$)	0.5	0.1 - 0.9, 0.1
Proportion perturbed samples in case group	1	-
Ratio of perturbed genes ($\|g_{pert}\|/\|G\|$)	0.1	0.1 - 0.9, 0.1
Shift β	2	-

B.2. Comparison with other methods for pathway-level DC

The performance of *DRaCOoN* was tested compared to eight other differential network reconstruction algorithms implemented in the *dcanr* R package [8] (see Table 1). A detailed description of the algorithms in the benchmark can be found below. The networks created by *DRaCOoN* were generated by using the Pearson, Spearman, and entropy co-expression metrics and splitting them according to whether the retrieved relationships were significant with respect to Δr, s or both.

The *dcanr* algorithms, similar to *DRaCOoN* mode 1, use DC analysis, but in our benchmark, we used them in a network-based DC method approach. To do so, we filter the outputs of the *dcanr* algorithms using our simulated network structures to exclusively evaluate their performance in network-based DC, thus inferring differential GRNs and providing a framework for assessing which method best identifies differential edges in a predefined network structure. We established a pipeline to apply each algorithm, with default parameters evaluated, to every simulation. By comparing the outcomes of each method to the corresponding ground truth network of each simulation, we computed performance in terms of the Matthews correlation coefficient (MCC) [35]. For those methods based on statistical testing, we selected significant interactions using the standard threshold of 0.01 over the BH-FDR-corrected p-values. This same criterion applied to the *DRaCOoN*-reconstructed threshold.

Z-score

Using Fisher's z-transformation, the *Z-score* algorithm by Zhang et al. [36] converts gene pair correlations to z-scores, which approximates a normal distribution. This transformation enables easier statistical significance testing. By calculating the difference between the z-scores and dividing it by the convoluted standard error, the algorithm accounts for sampling variability. The resulting value can be compared to a standard normal distribution under the null hypothesis of no difference between the population correlation coefficients.

DICER

DICER (Differential Correlation in Expression for meta-module Recovery) [27] is designed to detect co-expression modules and uses a permutation-based method to test for significant differences in correlations that make up these co-expression modules. To discover differential co-expression DICER first randomly assigns the samples' class labels and from this calculates a correlation normal distribution for each gene that combines the two classes. It examines whether a set of correlation scores was more likely to be sampled from the real distribution or the random normal distribution using a Bayesian statistical framework. The log-likelihood ratio between each gene pair's correlation coming from the genuine distribution and the random distribution is then calculated. The gene pair is seen to be considerably differential co-expressed when the ratio is positive, which denotes a considerable departure from the random distribution.

DiffCoEx

DiffCoEx [37] is an algorithm that finds differential co-expressed genes and modules by applying Weighted Correlation Network Analysis (WGCNA) [59] to a differential adjacency matrix representing the correlation change between conditions. For this it first creates correlation matrices for all gene pairs for each condition. Then differential matrices between these matrices are calculated in which entries are the differences between entries of the correlation matrices. After identifying differential co-expressed gene pairs, their topological overlap is calculated to see how the neighboring gene pairs

changed co-expression, resulting in an overlap matrix. This can be useful to see if both genes of the pair have correlation changes with the same group of genes [37]. This overlap matrix is used as input for hierarchical clustering to find differential expressed modules.

EBcoexpress

EBcoexpress [38] uses an empirical Bayesian approach to identify differential co-expression. For this a pairwise correlation matrix is computed using the standardized gene expression data for each condition resulting in partial correlation matrices. The algorithm can integrate multiple studies and multiple conditions. A Fisher Z-transformation is applied to the correlation matrices. This leads to a model which assumes that the correlations for a pair of genes across different conditions can be sampled from a mixture of multivariate Gaussians. The partial correlation matrices are used to estimate the parameters of these Gaussians, by using the expectation-maximization (EM) algorithm [60]. The empirical Bayes method is used to estimate the variance of the partial correlations, which is a weighted average of the sample variance and the variance of the prior distribution. The estimated partial correlations are then used to test for differential co-expression between two conditions. Specifically, the difference in the partial correlations between the two conditions is computed, and a Z-score is calculated by dividing the difference by the standard error of the difference. The Z-score is then used to compute a p-value for each gene pair using a normal distribution.

Entropy

Ho et al. [24] introduced an entropy-based measure for differential co-expression analysis using Shannon entropy. They found that when gene expression patterns are strongly correlated, the plot of expressions from one gene against another forms elongated ellipsoids. Analyzing the eigenvalues of the correlation matrix reveals that high correlation results in only a few significantly large eigenvalues. Normalizing and treating these eigenvalues as probabilities creates a distribution with a single highly probable outcome, representing the elongated axis. Entropy, which measures uncertainty, is highest when all values are equally likely and approaches zero when one outcome becomes certain. Hence, highly correlated genetic expressions exhibit low entropy. This entropy-based approach quantifies the variability of gene expression patterns and helps assess co-expression strength in differential co-expression analysis.

GGMs

The GGM (Graphical Gaussian Model) based approach to finding differential co-expression was introduced in 2011 by Chu et al. [39]. It uses these GGMs to quantify differential gene connectivity patterns between two disease states. The method involves estimating the covariance matrix and partial correlation matrix for each disease state, and then calculating the empirical posterior probability that the partial correlation between two genes is non-zero. The posterior probabilities are then used to calculate

the posterior odds ratio (postOR) for each edge in the network, which represents the likelihood of an edge being present in disease state versus the control state.

MAGIC

Modulated gene/gene set interaction (MAGIC) is an algorithm invented by Hsiao et al. [40] in 2016 as inference tool to detect gene interactions that are modulated by a third factors, such as transcription factor presence or more general cell conditions. The algorithm first filters the data by removing genes with low intensity after which correlation matrices of the two expression matrices are created. Both sets are then fisher-transformed to account for differences in sample sizes and a test is performed to estimate the statistical significance of the interaction. The test calculates a differential matrix of both correlation matrices by subtracting their absolute counterparts from one another. The hypothesis is that for a not modulated genes this differential should be zero, while for modulated genes it shouldn't. Since the data is normally distributed after a fisher transformation significance of the interaction can directly be assessed through computing the cumulative distribution function of the correlation matrix. After testing for significance the data is subjected to an inverse fisher transformation to compute a score reflective of the strength of the change in correlation.

FTGI

The FTGI ("Fast finding Three-way Gene Interactions") algorithm developed by Kayano et al. [41] in 2009 aims to detect three-way gene interactions in co-expression analysis. It utilizes linear discriminant analysis and correlation filtering techniques. The algorithm has multiple steps, the first of which determines if genes are differentially expressed between specific conditions. Then, it performs tests on gene pairs using correlation and discriminant analysis. If a gene pair can be distinguished between certain conditions, an interaction test is conducted using maximum likelihood estimation. Differential co-expression is identified if the likelihood difference surpasses a threshold.

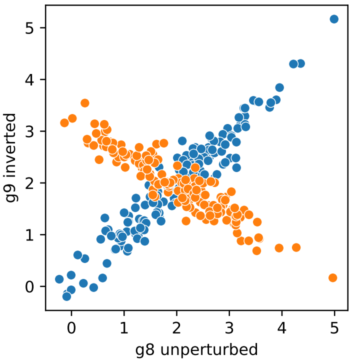

metric	control	case	Δr	s
entropy	0.100	0.154	0.054**	-0.533**
pearson	0.959	-0.929	1.887**	-0.242*
spearman	0.947	-0.920	1.867**	-0.118

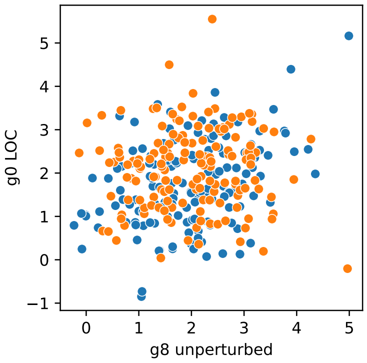

metric	control	case	Δr	s
entropy	0.620	0.693	0.073**	-0.018**
pearson	0.378	-0.001	0.379**	-0.005
spearman	0.340	0.051	0.289*	0.004

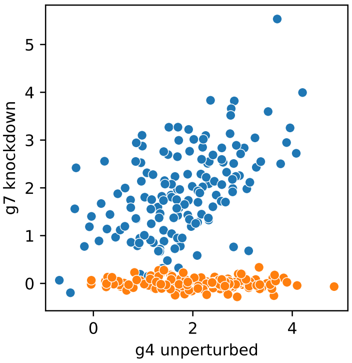

metric	control	case	Δr	s
entropy	0.540	0.691	0.150**	-0.075**
pearson	0.538	-0.072	0.610**	0.157
spearman	0.505	-0.060	0.564**	0.238*

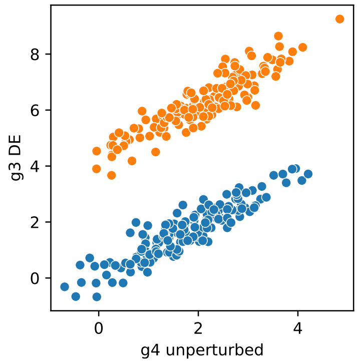

metric	control	case	Δr	s
entropy	0.183	0.186	0.003	-0.376**
pearson	0.911	0.908	0.002	0.406**
spearman	0.894	0.903	0.009	0.310**

● control ● case

Fig. B1: This example showcases the perturbation effects within a simulated gene regulatory network consisting of 10 genes and 3 transcription factors, with one gene affected by each perturbation type. Each subplot represents a scatterplot comparing the expression levels of a pair of genes under 'control' (blue) and 'case' (orange) conditions, indicating the presence of differential expression (DE), gene knockdown, inversion, or loss of co-expression (LOC) perturbations as noted. Below each scatterplot, a table displays the co-expression metrics ('entropy', 'pearson', and 'spearman') for each gene pair, together with the differential metrics Δr and s, with significant p-values ($p < 0.05$ indicated by *, $p < 0.01$ by **) obtained through the permutation test reflecting identified differential association relationships in the case condition.

Supplementary Results

C. Benchmark on simulated data by perturbation type

In Figure C2, we differently represent the results of Figure 3 by displaying methods' performance based on the perturbation type and aggregated across all ratios of case-to-control samples. The MCC scores for each algorithm under each perturbation type are averaged over the entire spectrum of case-to-control ratios, providing an overarching view of how each algorithm performs irrespective of the specific case-to-control ratio. This aggregation highlights the methods' capabilities to handle different types of biological perturbations within the simulated datasets of varying ratios of case samples to the total number of samples.

Most algorithms perform well with gene knockdown perturbations, which suggests that this perturbation type is easier to detect compared to others. *DRaCOoN* with entropy and $\Delta r + s$ show high median MCC values, indicating strong performance. For gene shifts, the performance of most algorithms is slightly lower than for gene knockdowns, but *DRaCOoN* methods still maintain robust performance. The loss of correlation perturbation appears to be the most challenging for the algorithms, as reflected by lower MCC scores across the board. The presence of outliers, as indicated by the dots outside of the boxplots, shows that there is some variability in performance within the methods for each perturbation type. Based on these results, the methods incorporated in the *DRaCOoN* suite, especially when using entropy-based measures, show strong and consistent performance across all types of perturbations, indicating a robustness that could be beneficial in real-world applications where multiple perturbation types are likely to occur.

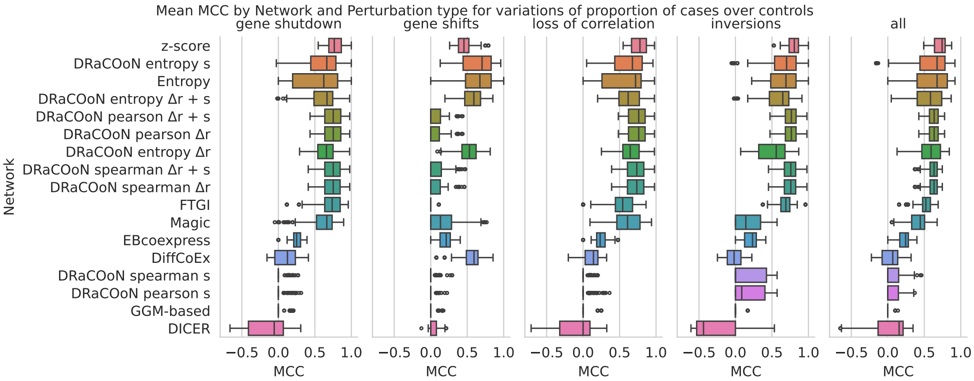

Fig. C2: MCC scores by network pathway-level DC method and perturbation type when varying the proportion of cases and controls. The distribution of MCC for various algorithms is faceted by perturbation type, including gene knockdown, gene differential expression (DE), loss of correlation (LOC), inversions, and a combination of all perturbations. The boxplots are ordered according to the mean MCC of each method across all perturbation types, highlighting the comparative robustness and sensitivity of the algorithms to the specific perturbation applied.

The insights in Figure 4 are presented differently in Figure C3, where the performance of the methods is aggregated over all ratios of perturbed genes depending on the type of perturbation. An overall picture of each method's performance, independent of the specific percentage of disturbed genes, is obtained by averaging the MCC scores for each algorithm under each type of perturbation. This aggregation technique demonstrates the method's ability to manage various biological perturbations in the simulated datasets with modified perturbed gene proportions, an important factor in a variety of biological and clinical contexts.

Figure C3 underscores the algorithms' capabilities to maintain prediction MCC as the proportion of perturbed genes changes, reflecting their potential application in various biological and clinical research settings. Gene knockdowns and shifts are notably better resolved by the algorithms, as evidenced by the higher MCC values, underscoring these methods' sensitivity to such perturbations. Conversely, the loss of correlation presents a significant challenge for all methods, indicated by lower MCC scores and greater score dispersion, reflecting a more complicated detection landscape. The variability in algorithmic performance is further accentuated by the presence of outliers, particularly in the aggregated 'all' perturbation category. Methods such as *GGM-based* and *DICER* lag in robustness, often yielding lower performance metrics, which may reflect a reduced capability to handle the diverse perturbations effectively. However, *DRaCOoN* methods using entropy as an association metric and s as a differential metric, together with *dcanr*'s entropy, still maintain good performance, indicating their ability to detect changes in gene expression levels. The presence of outliers and the spread of the interquartile range in the boxplots indicate variability in the performance of methods.

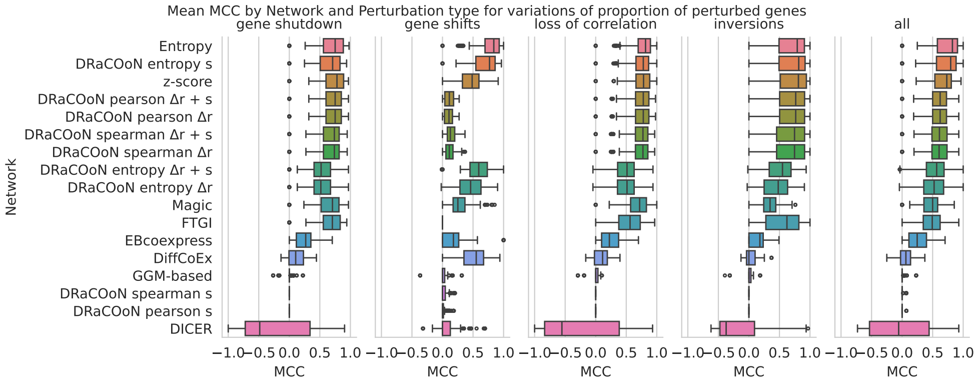

Fig. C3: Comparative Performance of Network Reconstruction Methods Across Different Perturbation Types when varying the proportion of perturbed genes. The distribution of MCC for various algorithms is faceted by perturbation type, including gene knockdown, gene shifts, loss of correlation, inversions, and a combination of all perturbations. The methods are sorted by their average MCC performance across all perturbation scenarios, offering a comparative perspective on their robustness and adaptability to each specific perturbation.

D. Validation in real datasets

D.1. Validation in a real dataset on bone healing

D.1.1. GSE99580 dataset description

For the evaluation of *DraCOoN*'s ability to reconstruct biologically significant networks, we used a subset of the GSE99580 gene expression dataset Hussein et al. [42] from the Gene Expression Omnibus (GEO) database [43]. The original experimental setup corresponds to a case-control study that evaluates the impact of phosphate deficiency on bone healing in mice [42, 61]. Specifically, we selected 126 control samples from three male mouse strains (A/J, C57BL/6J, and C3H/HeJ), which represented standard bone healing conditions in mice without any dietary alterations. This approach allowed us to focus on the natural gene expression patterns associated with bone repair under normal conditions to construct biologically relevant gene networks.

The fracture calluses were collected at various postoperative days (3, 5, 7, 10, 14, 18, 21, 28, and 35), and then gene expression was analyzed using microarrays (Affymetrix Mouse Gene 1.0 ST Array). The number of samples available for each time point is shown in Table D2.

Table D2: Collection of Fracture Calluses and number of samples analyzed at various postoperative days using microarrays for the GSE99580 dataset. All samples after the time point of day 0 correspond to days post-operation.

Time point	Number of Samples
0 (Before fracture)	13
3	9
5	9
7	13
10	17
14	9
18	9
21	13
28	21
35	13

In this case, we retrieve the preprocessed time series dataset from Gene Expression Omnibus, where raw Affymetrix CEL files were normalized to yield Entrez Gene-identifier-specific expression values using the implementation of the Robust Multiarray Average (RMA) in the Affy R package [62]. Further preprocessing details can be found in Gene Expression Omnibus under entry GSE99580.

D.1.2. Gene ID mapping between TRRUST V2 and GSE99580

To run pathway-level DC with *DRaCOoN* (mode 2), we used the TRRUST V2 database [33], a carefully curated collection of transcriptional regulatory relationships unraveled by sentence-based text mining. This database provides the established *Mus musculus* TFs-TGs regulatory network (2456 nodes and 7057 relationships, release note: 2018.04.16), which we used as input for *DRaCOoN*.

In order to properly map the genes analyzed in the original study (GSE99580) and the genes present in the TRRUST V2 database, we used the *MyGene.info* Python package [44]. Since TRRUST V2 uses gene symbols to refer to genes and the GSE99580 data set used Affymetrix IDs, we first converted TRRUST V2 symbols to Entrez IDs. The conversion rate was 99.6%, as 10 genes couldn't be properly mapped, resulting in 2446 genes in 7036 relationships (we also removed relationships involving the 10 unmappable genes). Following this, the 21046 Affymetrix IDs in GSE99580 were converted to Entrez IDs (100% conversion rate), and the intersubsection between these and the TRRUST V2 converted Entrez IDs was estimated. This way, we continued the analysis with 2378 overlapping IDs encompassed in 6290 TRRUST V2 relationships. Such relationships, together with the GSE99580 subset for the 2378 mapped gene IDs, were used as input for *DRaCOoN*'s pathway-level DC analysis.

D.1.3. Time-resolved differential expression analysis

As a comparison for subsequent pathway-level DC analysis, we performed classical differential expression analysis (DEA) using the *limma* R package [63]. Only the 2378 genes from the original dataset that are also present in TRRUST V2 were tested for differential expression. We obtained differentially expressed genes (DEGs), marked in red in Figure D4, using FDR-corrected p-values< 0.01. Some plots show a wide range of fold changes (e.g., day 0–day 5), indicating substantial upregulation and downregulation of genes. Others, particularly towards later days, show a more concentrated range of fold changes, suggesting a more muted response or a return to baseline expression levels. The initial response (day 0–day 3) shows a moderate number of DEGs, which significantly increases by day 5 and day 7, implying an active biological response or adaptation occurring during these periods. Over time, the number of DEGs and the magnitude of expression changes decrease, which could indicate a resolution of the response or an adaptation of the system to the new conditions.

We used the *clusterProfiler* R package [64] to run over-representation analysis (ORA), based on gene ontology (GO) terms, over the DEGs using a standard q-value cutoff of 0.01 and the 2378 mapped genes as background. Figure D5 shows the results of the ORA for DEGs across various time points or conditions, compared to day 0. During the initial postoperative days, there is noticeable involvement of genes associated with the cell cycle, cell adhesion, angiogenesis, blood vessel development, and morphogenesis. This early response is crucial for initiating bone repair, with angiogenesis and blood vessel development being particularly important for delivering necessary nutrients and cells to the fracture site [65]. By Day 7, there is a transition towards the regulation of lymphocyte and leukocyte activation, along with continued emphasis on cell adhesion and blood vessel morphogenesis. The regulation of immune system

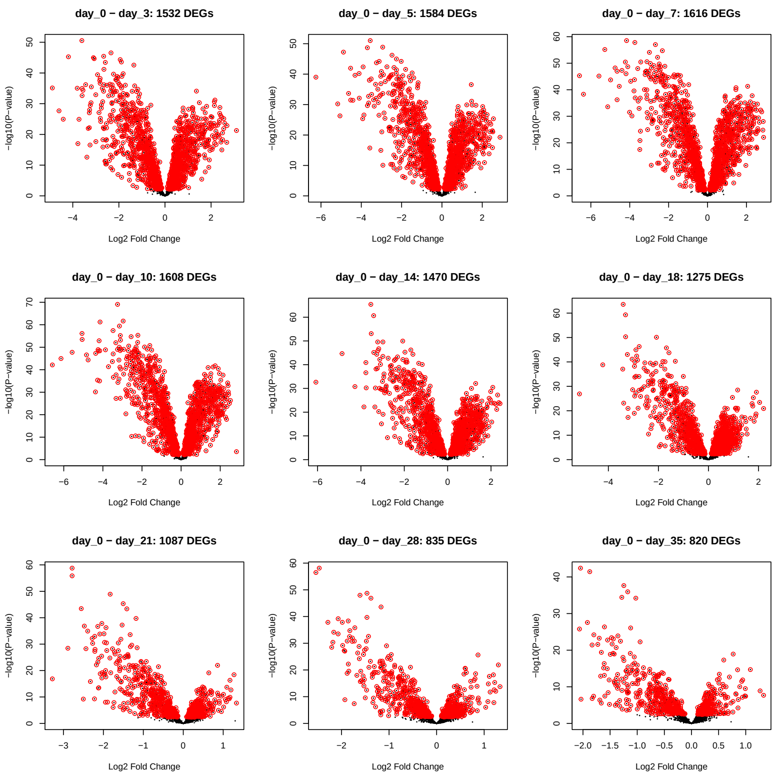

Fig. D4: Volcano plots represent the differential expression analysis for the nine different time point comparisons. Each subplot corresponds to one of these comparisons, visualizing the relationship between the log fold change (x-axis) and the negative logarithm of the FDR-corrected p-value (y-axis) for each gene. Genes highlighted in red represent those that are significantly differentially expressed, meeting the criteria of a p-value below 0.01.

processes indicates a robust immune response, likely involved in clearing debris and combating potential infections, which is a critical aspect of bone healing [66]. As the healing progresses, there is an observable transition to processes like DNA metabolic processes, ossification, and the regulation of angiogenesis and vasculature development. These changes suggest a move towards bone remodeling and maturation, with ossification being a key step in bone hardening and final repair. Over time, the gene expression changes decrease, indicating a resolution of the active repair process and a gradual return to normal bone physiology. Noticeably, no BP GO term was enriched for the DEGs obtained in the comparison between day 0 and day 35.

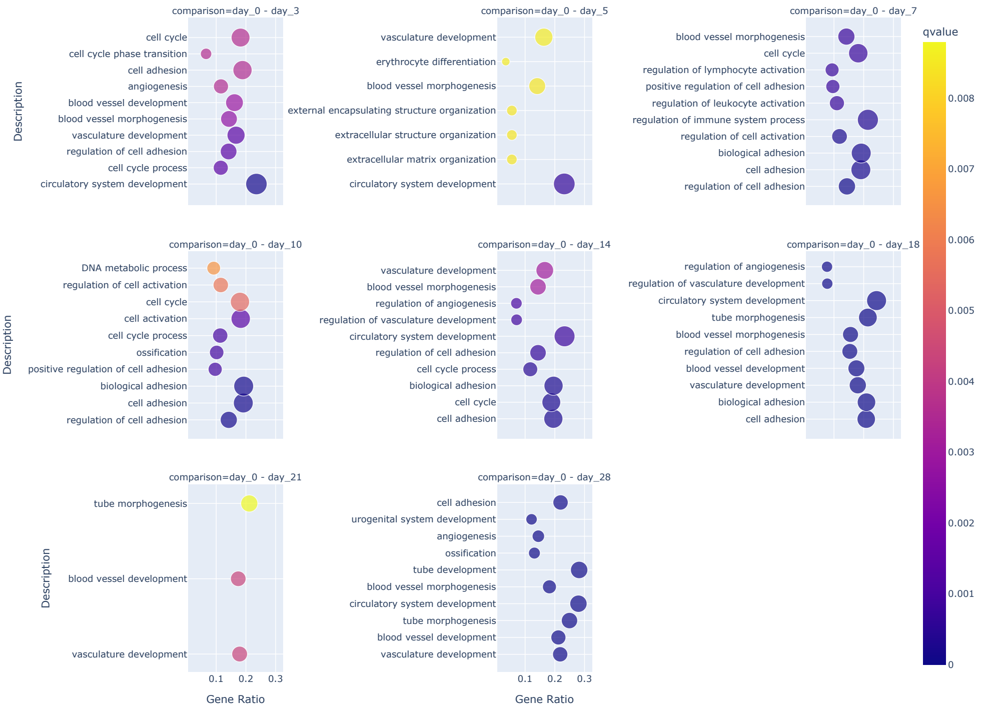

Fig. D5: Dot plot illustrating the results of gene ontology's biological processes (BP) over-representation tests (ORA) across the various time points for the previously-identified DEGs in the bone healing dataset. Each subplot corresponds to a specific comparison, with the x-axis representing the gene ratio and the y-axis detailing the description of the identified BP. The size of each point is proportional to the count of genes in each set, indicating the relative magnitude of the gene set. The color of the points represents the q-value (FDR-corrected p-value), with a color gradient indicating the level of significance, where darker hues correspond to more significant gene sets. This visualization highlights the enriched BPs in each comparison, providing insights into the dynamic changes in gene expression and biological functions across different time points or experimental conditions. For representation purposes, we show the top 10 most significant GO-terms of each comparison according to q-value.

D.1.4. Running DRaCOoN

Using this time-resolved gene expression data, we applied *DRaCOoN* to infer differential regulatory (DR) networks to highlight the differences between day 0 and the subsequent days, with the goal of examining the dynamic changes in gene regulation throughout bone healing. As with simulated expression datasets, the networks generated by *DRaCOoN* were created using the three association metrics (Entropy,

Pearson's, and Spearman's) and then split based on whether the retrieved relationships were significant according to Δr, s, or both. We selected significant interactions for the differential metrics if their FDR-BH adjusted p-value was < 0.01. In the case of the combined approach, we selected all relationships whose Δr or s FDR-BH-corrected p-value < 0.01.

D.1.5. *dcanr's* algorithm implementation in real-world large datasets

The implementation details provided in the *dcanr* package reveal several aspects that could impact its performance with large amounts of data. For instance, the use of matrix operations, especially conversion between matrices and vectors, involves creating copies of data and potentially manipulating large matrices. This can be memory-intensive for large datasets. The permutation test implementation uses a *foreach* loop for permutations. While *foreach* can be parallelized, the default setting without parallel back-end registration will run sequentially, leading to longer run times for large datasets. The computational cost also increases with the number of permutations and the size of the data.

We ran *dcanr* for large datasets as follows:. Given the complexity and size of the GRN at the TRRUSTV2 database [33], our analysis focused on the examination of distinct subnetworks. For each subnetwork, differential co-expression analysis was performed to identify changes in gene-gene interactions under different experimental conditions. We assessed the statistical significance of observed changes in co-expression within each subnetwork, as implemented in the *dcanr* package. This approach generated raw p-values for each gene pair, indicating the likelihood that observed changes in co-expression occurred by chance. After combining the results from the multiple comparisons inherent in analyzing numerous gene pairs, we employed a correction for multiple testing (FDR-BH).

D.1.6. Size of the reconstructed networks

Figure D6 shows a comparative analysis of network sizes over time using DEGs and DEG-networks as references. The node sizes labeled as 'DEGs' represent the number of DEGs, while the edge sizes labeled as 'DEGs' correspond to the number of edges in the TRRUST V2 database, whose both nodes are also present in the original GSE99580 dataset (see D.1.2 for mapping) and that contained at least one DEG. Reconstructing networks using DEGs consistently results in much larger networks (either by nodes or edges), followed by those networks reconstructed using the entropy-based association metric with the s differential metric, sometimes in combination with Δr.

D.1.7. Extended ORA over nodes in the reconstructed networks per time point comparison

The process of bone healing involves complex biological mechanisms that are crucial for the repair and regeneration of bone tissue following injury. Several GO terms related to bone healing have been identified as significant for understanding the molecular and cellular processes involved. These include ossification (GO:0001503),

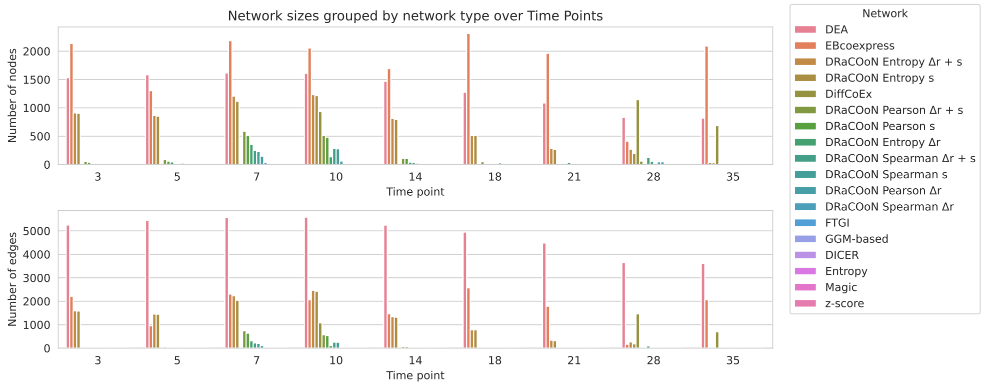

Fig. D6: Bar plot illustrating network sizes for different methods across the nine time points, measured by the number of nodes (top) and the number of edges (bottom). The networks are ordered by their average size over all time points, highlighting the methods that capture more interactions (nodes). The legend is sorted according to the mean size of networks, providing a reference to the largest networks.

bone development (GO:0060348), wound healing (GO:0042060), response to wounding (GO:0009611), extracellular matrix organization (GO:0030198), skeletal system development (GO:0001501), cartilage development (GO:0051216), and osteoblast differentiation (GO:0001649) [57]. These terms cover a broad range of biological activities from the initial response to injury, through the stages of bone repair, to the final remodeling and strengthening of the healed bone. Again, we used Fisher's exact test as described in subsection 3.2.1, a standard Adj. p-value cutoff of 0.01, and the 2378 mapped genes as background. We filtered the results for the GO terms mentioned above an the results are shown in Figure D8.

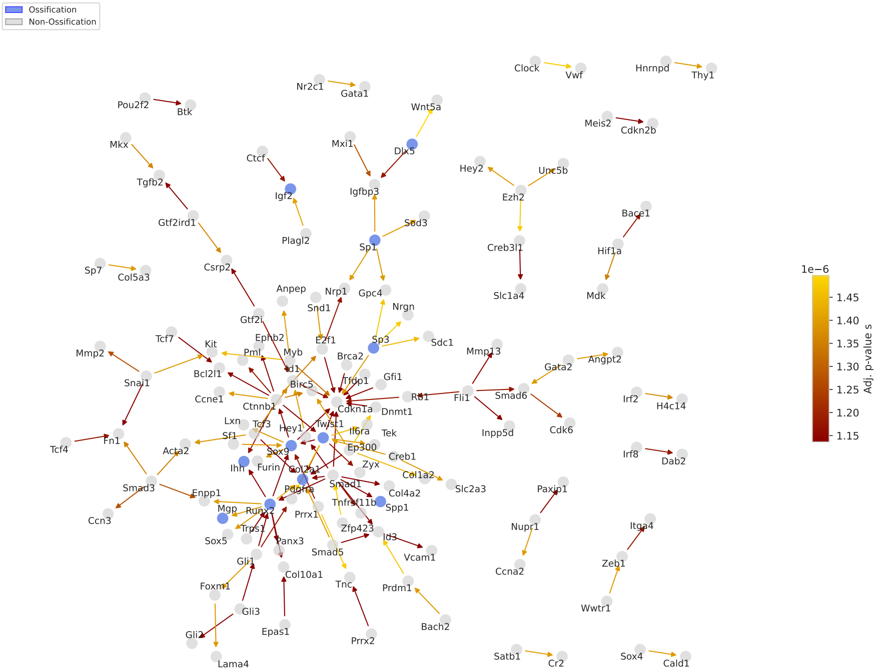

Fig. D7: Reconstructed DGRN for the comparison at time point 10, using the entropy association metric and the s differential metric (Adj. p-value < 0.0000015) . Ossification-related genes (nodes) are highlighted in blue and other genes in grey. Edges represent differential TF-TG interactions, with the color gradient indicating the adjusted p-value of the s metric, suggesting the strength and significance of the regulatory effect. This visualization captures the intricate web of significant differential TF-TG relationships, underscoring the critical role of ossification-related genes within the network.

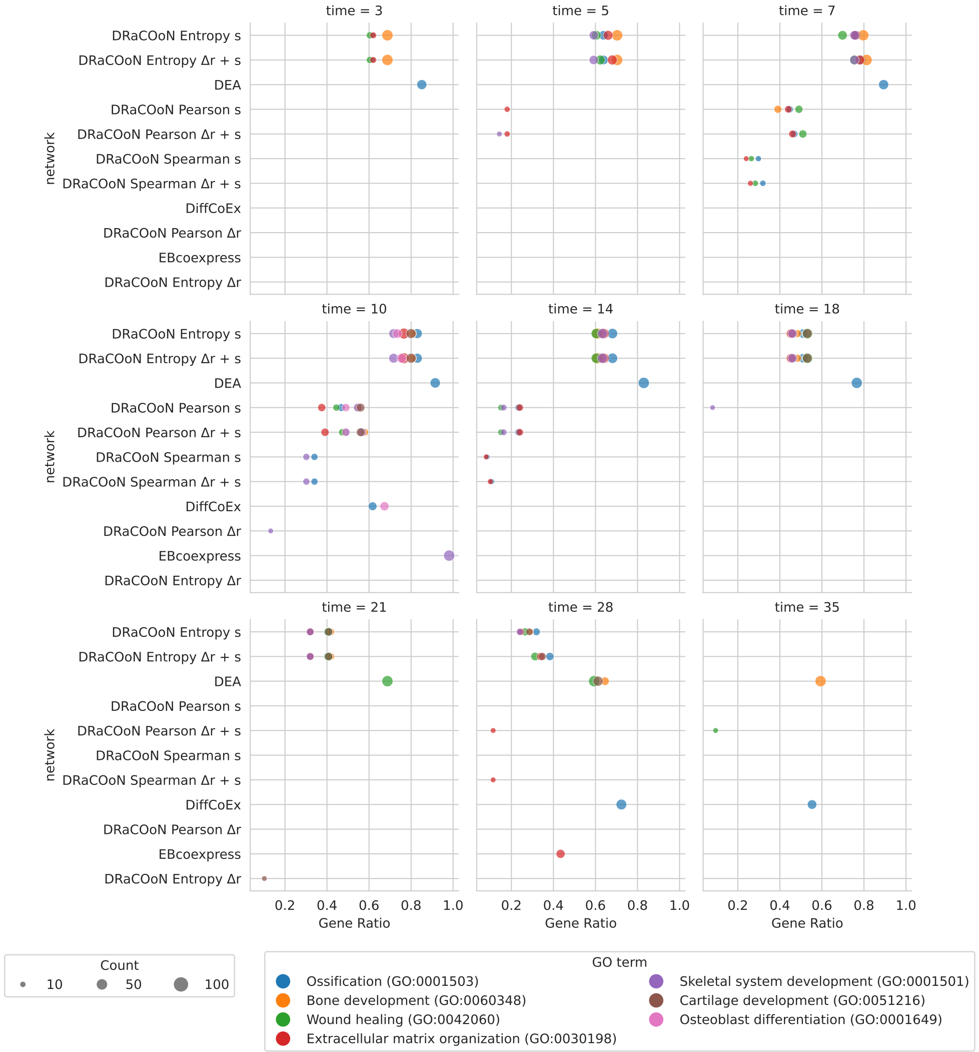

Fig. D8: Time-resolved GO-based ORA across various post-operative days following bone fracture in mice. Each dot plot represents a distinct time point comparison, with the x-axis indicating the gene ratio involved in the identified significant GO terms and the y-axis listing the network analysis methods. Points are colored according to the specific GO term 'Description'. Marker size corresponds to the 'Count' of genes associated with each GO term, highlighting the relative abundance of significant genes within each category. Only networks exhibiting significant GO enrichment (FDR-BH Adj. p-value) are represented.

D.2. Validation in a real dataset on colorectal cancer (CRC)

D.2.1. GSE89076 dataset description

For the evaluation of *DraCOoN*'s ability to reconstruct differential gene regulatory networks, we used the GSE89076 gene expression dataset [48] from the Gene Expression Omnibus (GEO) database [43]. This dataset comprises a case-control study investigating differences in gene expression between colorectal cancer and normal tissues in humans, using a paired design where available. The dataset consists of 80 samples and is primarily paired with tumor and normal tissue samples obtained from the same patient. However, two patients (4 and 5) have two tumor samples each and a corresponding normal sample instead of the expected single tumor sample. We therefore proceeded with an unpaired analysis, comparing all 41 tumor samples to all 39 normal samples.

DNA microarray analysis was performed on tissue specimens using the Agilent-039494 SurePrint G3 Human GE v2 8x60K Microarray 039381 platform. The specimens were collected from various locations within the colon and rectum, including the sigmoid colon, rectum, cecum, and ascending colon, reflecting the heterogeneity of colorectal cancer.

We utilized the preprocessed dataset available from GEO. The original raw data files were background-corrected using the *normexp* method with an offset of 50 and then normalized using quantile normalization. Both methods were implemented within the *limma* R package [63]. The final analyzed dataset comprised 41 tumor samples and 39 normal samples.

D.2.2. Gene ID mapping between TRRUST V2 and GSE89076

To perform pathway-level DC with *DRaCOoN* (mode 2), we used the TRRUST V2 database [33], a carefully curated collection of transcriptional regulatory relationships. This database provides the established human TFs-TGs regulatory network (2862 nodes and 9396 relationships, release note: 2018.04.16), which we used as input for *DRaCOoN*.

To correctly map the genes analyzed in the original study (GSE89076) and the genes present in the TRRUST V2 database, we used the *MyGene.info* Python package [44]. Since TRRUST V2 uses gene symbols to refer to genes, and the GSE89076 dataset uses Agilent probe IDs, we first converted TRRUST V2 symbols to Entrez IDs. The conversion rate was 99.96%, resulting in 2853 genes in 9396 relationships (we also removed relationships that included the unmappable genes). Subsequently, the Agilent IDs in GSE99580 were converted to Entrez IDs (87.6% conversion rate), and the intersubsection between these and the TRRUST V2 converted Entrez IDs was estimated. Notably, a large proportion of the probes in these Agilent IDs were control probes that do not encode biological transcripts. In this way, we continued the analysis with 2531 overlapping IDs that were included in 7071 TRRUST V2 relationships. Such relationships, together with the GSE99580 subset for the 2531 mapped gene IDs, were used as input for the *DRaCOoN* pathway-level DC analysis.

D.2.3. Differential expression analysis: neoplasic vs. reference tissue

As a comparison for subsequent pathway-level DC analysis, we performed classical differential expression analysis (DEA) using the *limma* R package [63]. We performed differential expression analysis to identify genes with significantly altered expression levels between the tumor and normal tissue groups, using the 2531 genes from the original dataset that are also present in the filtered TRRUST V2 database. We obtained differentially expressed genes (DEGs) using an FDR-corrected p-value < 0.01.

Figure D9 shows the volcano plot representing the DEA results for the comparison between tumor (neoplastic) and normal (reference) tissues. The plot reveals a substantial number of genes with altered expression levels in tumor tissues compared to normal tissues. Many genes exhibit a log2 fold change greater than 2 (both positive and negative), suggesting a strong biological effect.

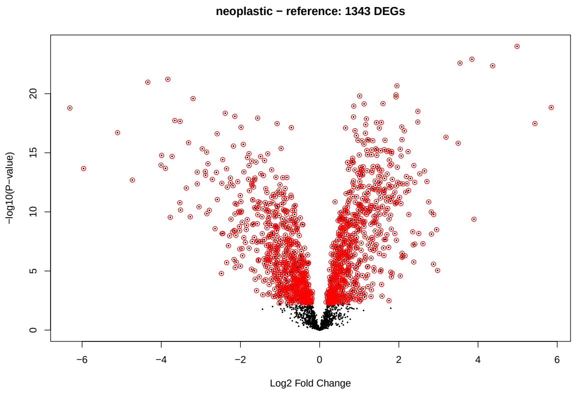

Fig. D9: Volcano plot representing the differential expression analysis for the comparison between tumor and normal tissues in the GSE89076 dataset. The plot visualizes the relationship between the log fold change (x-axis) and the negative logarithm of the FDR-corrected p-value (y-axis) for each gene. Genes highlighted in red represent those that are significantly differentially expressed, with an FDR-corrected p-value below 0.01.

We used the *clusterProfiler* R package [64] to run over-representation analysis (ORA), based on gene ontology (GO) Biological Process terms, over the DEGs using a standard q-value cutoff of 0.01 and the 2531 mapped genes as background. Figure D10 shows the results of the ORA for DEGs, comparing tumor tissue to normal tissue. Several biological processes (BP) were found to be significantly enriched among the DEGs identified in the CRC dataset. As shown in Figure D10, the most prominent enriched terms are strongly associated with fundamental cellular processes related to DNA metabolism, cell cycle, and chromosome management. Specifically, terms like "cell cycle process", "DNA metabolic process", "DNA repair", "DNA-dependent DNA

replication", "chromosome organization", and "cellular response to DNA damage stimulus" are highlighted. Additionally, processes involved in the production of cellular machinery, such as "ribosome biogenesis" and "ribonucleoprotein complex biogenesis", are also significantly enriched. These findings point towards heightened cellular proliferation, active DNA replication and repair mechanisms, and increased biosynthetic activity, which are characteristic hallmarks of cancerous tissues compared to normal reference tissues.

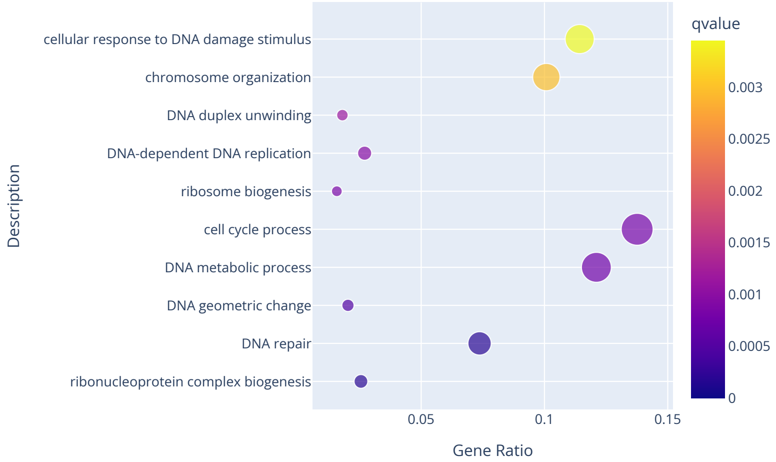

Fig. D10: Dot plot illustrating the results of Gene Ontology Biological Process (GO BP) over-representation tests for the identified DEGs in the GSE89076 dataset (tumor vs. normal). The x-axis represents the gene ratio, and the y-axis details the description of the identified BP. The size of each point is proportional to the count of genes in each set, indicating the relative magnitude of the gene set. The color of the points represents the q-value (FDR-corrected p-value), with the color scale shown on the right (darker colors indicate higher significance/lower q-values). For representation purposes, we show the top 10 most significant GO terms according to q-value.

D.2.4. *DRaCOoN* and *dcanr's* algorithm implementation in the CRC dataset

Using the GSE89076 preprocessed dataset, we applied *DRaCOoN* to infer differential regulatory (DR) networks to highlight the differences between tumor and healthy tissues, with the goal of examining the dynamic changes in gene regulation upon CRC. As with simulated expression datasets, the networks generated by *DRaCOoN* were created using the three association metrics (Entropy, Pearson's, and Spearman's) and then split based on whether the retrieved relationships were significant according to Δr, s, or both. We selected significant interactions for the differential metrics if their

FDR-BH adjusted p-value was < 0.01. In the combined approach, we considered each relationship to be differentially co-expressed if either its Δr or s had a FDR-BH-corrected p-value < 0.01.

The implementation details provided in the *dcanr* package reveal several aspects that could impact its performance with very large amounts of data. For instance, the use of matrix operations, especially conversion between matrices and vectors, involves creating copies of data and potentially manipulating large matrices. This can be memory-intensive for large datasets. The permutation test implementation uses a *foreach* loop for permutations. While *foreach* can be parallelized, the default setting without parallel back-end registration will run sequentially, leading to longer run times for large datasets. The computational cost also increases with the number of permutations and the size of the data.

Similar to the previous example, to enable the application of *dcanr* to the large TRRUST V2 GRN (7071 relationships among 2531 genes), we divided the network into smaller subnetworks for computational feasibility. Specifically, we split the TRRUST V2 network into chunks of 100 gene-gene relationships, based on the order in which they appear in the TRRUST V2 file. For each subnetwork, we then performed differential co-expression analysis using *dcanr*, generating raw p-values for each gene pair. We obtain the differentially coexpressed network by the intersubsection of significant interactions according to *dcanr* and present in the TRRUST V2 network. After combining the results from the multiple comparisons inherent in analyzing numerous gene pairs, we employed a correction for multiple testing (FDR-BH).

D.2.5. Size of the reconstructed networks

We applied *DRaCOoN* to the GSE89076 dataset to infer differential regulatory (DR) networks, highlighting the differences between colorectal cancer and normal tissue. The methodology for running *DRaCOoN* on this dataset followed the procedures outlined in subsection D.1.4. Specifically, we employed the three association metrics (Entropy, Pearson, and Spearman) to generate networks and subsequently split these networks based on the significance of the relationships as determined by Δr, s, or both. Significant interactions for the differential metrics were selected based on an FDR-BH adjusted p-value threshold of < 0.01. For the combined approach, interactions were deemed significant if either their Δr or s FDR-BH-corrected p-value was < 0.01. Similarly, the *dcanr* algorithm was run on the GSE89076 dataset following the implementation details described in subsection D.1.5. As shown in Figure D11, the DiffCoEx network exhibits the largest size, followed by DEA and the DRaCOoN networks using entropy-based metrics and *dcanr*'s Entropy.

D.2.6. Comparison of Functional Enrichment Profiles: DEG vs. Differential Network (Entropy Δr)

Following the individual Over-Representation Analyses (ORA) for the Colorectal Cancer (CRC) dataset GSE89076, we conducted a direct comparison between the functional profiles derived from standard Differential Expression Analysis (DEA) and pathway-level differential network analysis. Specifically, we compared the ORA results

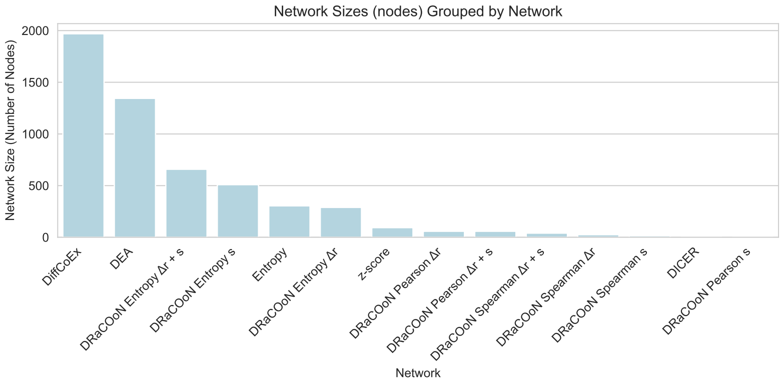

Fig. D11: Network sizes (number of nodes) across different network inference algorithms applied to the GSE89076 dataset. The bar plot displays the number of nodes included in each network, grouped by algorithm. Bars are sorted in descending order based on the average network size for each algorithm.

for the DEGs (Section D.2.3) with the ORA results for genes involved in significant differential interactions identified by *DRaCOoN* using the Entropy association metric and the Δr differential metric (Section D.2.4). The goal was to elucidate potentially different biological insights captured by feature-level versus interaction-strength-change analysis.

Figure D12 juxtaposes the top 10 significantly enriched Gene Ontology Biological Process (GO BP) terms for each approach. The left panel represents the enrichment from the DEG list, while the right panel shows the enrichment derived from the genes within the *DRaCOoN* Entropy Δr network.

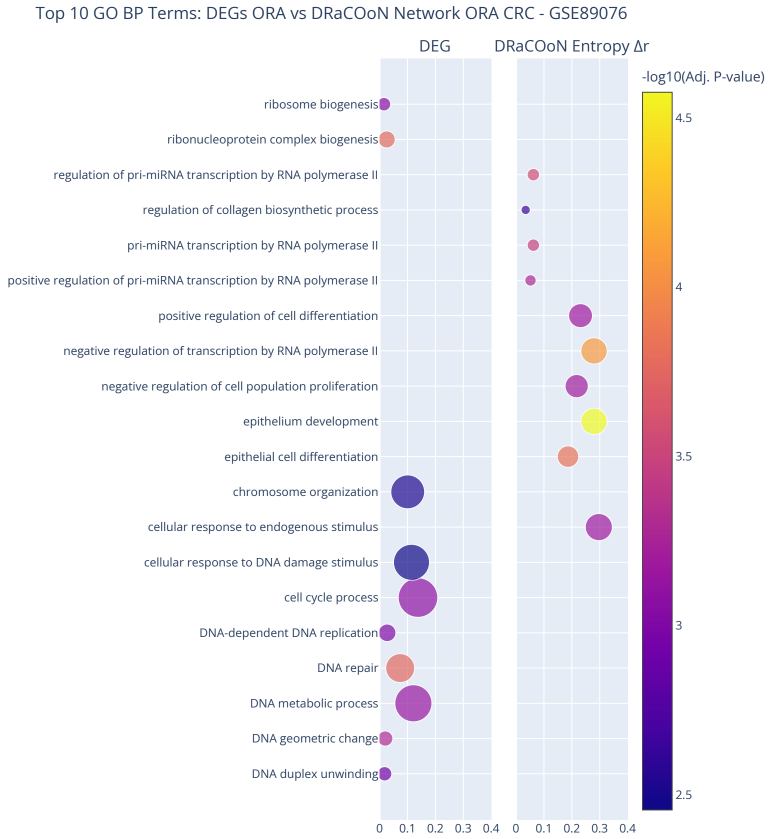

Fig. D12: Comparative Gene Ontology Biological Process (GO BP) enrichment dot plot for the CRC dataset (GSE89076, tumor vs. normal). Left panel: Top 10 enriched terms for DEGs identified via standard differential expression analysis. Right panel: Top 10 enriched terms for genes involved in significant differential interactions identified by *DRaCOoN* (Entropy Δr configuration). X-axis: Gene Ratio. Y-axis: GO BP Term. Point size: Gene Count. Color: -log10(Adjusted P-value), brighter/higher values indicate greater significance.

The comparison in Figure D12 highlights distinct functional emphases between the two analytical strategies. The DEG analysis (left panel) strongly enriches for fundamental processes associated with cell proliferation and genome maintenance, such as "cell cycle process", "DNA metabolic process", "DNA repair", "DNA-dependent DNA replication", and "chromosome organization". This profile reflects the generally heightened mitotic and metabolic activity characteristic of cancer cells identified through individual gene expression changes.

Conversely, the ORA based on the *DRaCOoN* Entropy Δr network (right panel) reveals a different set of enriched biological themes. While there is some overlap (e.g., "cellular response to DNA damage stimulus", "chromosome organization"), the network analysis prominently features terms related to the regulation of transcription, particularly by RNA polymerase II (e.g., "regulation of pri-miRNA transcription...", "negative regulation of transcription..."), along with processes involved in development and differentiation, such as "epithelium development", "epithelial cell differentiation", and "positive regulation of cell differentiation". The significance levels for these terms are notably high.

This contrast suggests that while DEA effectively captures the broad signatures of increased proliferation and DNA-related activity in CRC based on individual gene changes, the differential network analysis focusing on changes in interaction strength (Δr) using the Entropy metric preferentially identifies alterations in specific regulatory programs, particularly transcriptional control and developmental/differentiation pathways. This interaction-centric view may provide deeper insights into the dysregulated signaling and control mechanisms underlying the CRC phenotype, complementing the view obtained from analyzing individual gene expression levels alone.

D.3. Validation in a real dataset on head and neck squamous cell carcinoma (HNSCC)

D.3.1. GSE173855 Dataset Description

To validate *DraCOoN*'s performance on real-world data, we utilized the GSE173855 dataset [52], a transcriptomic study of Head and Neck Squamous Cell Carcinoma (HNSCC), obtained from the Gene Expression Omnibus (GEO) database [43]. This dataset contains RNA sequencing data from paired primary and relapsed HNSCC tumor samples. The primary objective of the original study was to investigate gene expression differences between primary and relapsed tumors, particularly regarding changes in transcriptional subtypes.

The original dataset, as described in Weber et al. [52], comprises 68 samples, representing 34 matched pairs of primary and relapsed tumors. However, the publicly available raw count data obtained from GEO for our analysis differed slightly, as detailed below. RNA-Seq was performed in the original study using the Illumina HiSeq 2000 platform, with cDNA libraries prepared for transcriptomic analysis. Critically, the original study used both whole-transcriptome sequencing and 3' mRNA sequencing on FFPE samples, incorporating specific protocols to address FFPE-related degradation. Furthermore, the published study utilized data from defined tumor regions and incorporated information from single-cell experiments for tumor subtype classification (classical, basal, and inflamed-mesenchymal) [52].

The raw count data, aligned to the GRCh38.p13 reference genome using the NCBI annotation, were downloaded from GEO. We loaded and merged the raw count files into a single matrix. Initial data exploration revealed that two samples present in the downloaded count matrix were missing from the provided metadata. These samples were excluded from further analysis.

The dataset comprises 68 samples, representing 34 matched pairs of primary and relapse tumors from individual patients. RNA-Seq was performed using the Illumina HiSeq 2000 platform, with cDNA libraries prepared for transcriptomic analysis. The samples were sequenced, and the resulting raw count data (aligned to the GRCh38.p13 reference genome using the NCBI annotation) were downloaded from GEO. We loaded and merged the raw count file into a single matrix.

Initial data exploration revealed that seven samples present in the metadata were missing from the count matrix, leading to a discrepancy in the number of samples between the two data sources. These samples were excluded from the count matrix. The remaining 61 samples were used for downstream analyses after merging. The final analyzed dataset consisted of 29 primary tumor samples and 32 relapse tumor samples.

D.3.2. Data Preprocessing and Normalization

We performed a series of preprocessing and normalization steps to prepare the raw count data for differential gene regulatory network analysis. All analyses, including data import, preprocessing, and normalization, were performed using custom Python scripts, leveraging R functions from the ‘edgeR‘ [67] and ‘limma‘ [63] Bioconductor packages.

First, we filtered low-expressed genes to reduce noise and improve the reliability of downstream analyses. Genes were retained if they had a counts-per-million (CPM) value greater than 1 in at least 29 samples (the size of the smallest group). This ensured that genes with consistently low expression across a significant portion of the samples were removed. This filtering step reduced the number of genes from 39,376 to 25,064.

Next, we normalized the filtered count data using the trimmed mean of M-values (TMM) method [68] to account for differences in library size and RNA composition between samples, providing a more accurate representation of relative gene expression.

D.3.3. Gene ID mapping between TRRUST V2 and GSE173855

To enable pathway-level differential co-expression analysis using *DraCOoN* (mode 2), we integrated the GSE173855 dataset with the TRRUST v2 database [33], a curated resource of human transcriptional regulatory relationships. The TRRUST v2 database (release note: 2018.04.16) contains 9,396 regulatory interactions between transcription factors (TFs) and their target genes (TGs), encompassing 2,862 unique genes represented by gene symbols.

A critical step was to establish a common identifier space between the gene expression data (GSE173855) and the TRRUST v2 network. The GSE173855 dataset used Entrez Gene IDs, while TRRUST v2 primarily uses gene symbols. We utilized the ‘MyGene.info‘ Python package [44] to facilitate the mapping process.

Next, we identified the intersubsection between the Entrez IDs present in the preprocessed GSE173855 expression data and the mapped Entrez IDs from TRRUST v2. This resulted in 2,522 common genes. We filtered the TRRUST v2 relationships to include only those interactions where both the TF and TG were among these 2,522 common genes and removed any duplicate TF-TG pairs. This final, filtered TRRUST v2 network contained 7,181 unique TF-TG relationships among 2,456 unique genes.

Finally, we subsetted the normalized GSE173855 expression matrix to include only the 2,456 genes present in the filtered TRRUST v2 network. This gene-matched expression matrix, along with the filtered TRRUST v2 network, served as input for the *DraCOoN* pathway-level differential co-expression analysis.

D.3.4. Differential Expression Analysis

To complement the subsequent differential co-expression analysis, we performed a standard differential expression analysis (DEA) using the ‘limma‘ R package [63]. This analysis aimed to identify genes with statistically significant changes in expression levels between relapse and primary HNSCC tumors, using the normalized expression data for the 2,456 genes common to both the GSE173855 dataset and the filtered TRRUST v2 network.

We constructed a design matrix representing the two conditions (relapse and primary) and used it to fit a linear model to the expression data. We employed the ‘voom‘ transformation [69] within ‘limma‘ to account for the mean-variance relationship in RNA-Seq data. Empirical Bayes moderation was applied using ‘eBayes‘ to improve the estimation of gene-wise variances. Following the significance cutoff of the original

publication by Weber et al. [52], differentially expressed genes (DEGs) were identified using an FDR-adjusted p-value threshold of 0.05 and no log2 fold-change (logFC) cutoff. We identified 133 DEGs, as shown in Figure D13.

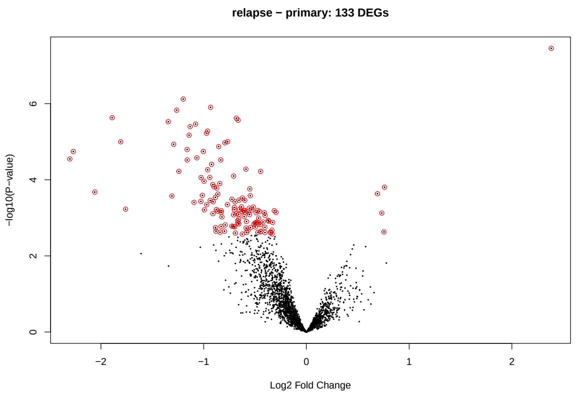

Fig. D13: Volcano plot of differential expression analysis results for the GSE173855 dataset (relapse vs. primary). The x-axis represents the log2 fold change (logFC), and the y-axis represents the negative base-10 logarithm of the FDR-adjusted p-value. Red points indicate genes considered significantly differentially expressed (adjusted p-value < 0.05).

To gain insights into the biological functions associated with the DEGs, we performed an ORA using the `clusterProfiler` R package [64]. The ORA was conducted using GO Biological Process (BP) terms. We used the set of all 2,456 genes present in the analysis as the background gene set and the identified DEGs as the foreground set. A q-value (FDR-adjusted p-value) cutoff of 0.05 was used to determine significant enrichment. As depicted in Figure D14, the most significantly enriched GO terms are predominantly related to immune cell processes, particularly activation, differentiation, and migration. Key terms include "T cell differentiation", "lymphocyte migration", "adaptive immune response", "leukocyte differentiation", "lymphocyte differentiation", and "T cell activation". This points towards substantial changes in the immune landscape and cellular activity between primary and relapsed HNSCC tumors.

D.3.5. *DRaCOoN* and *dcanr's* algorithm implementation in the HNSCC dataset

We applied *DRaCOoN* to the preprocessed GSE173855 HNSCC dataset to identify differential regulatory (DR) networks, highlighting the regulatory differences between relapse and primary tumor samples. The analysis utilized the normalized expression data (log2-CPM) for the 2,456 genes common to both the GSE173855 dataset and the filtered TRRUST v2 network (as described in subsection D.3.3). The filtered TRRUST v2 network (7,181 TF-TG relationships) served as the structural prior.

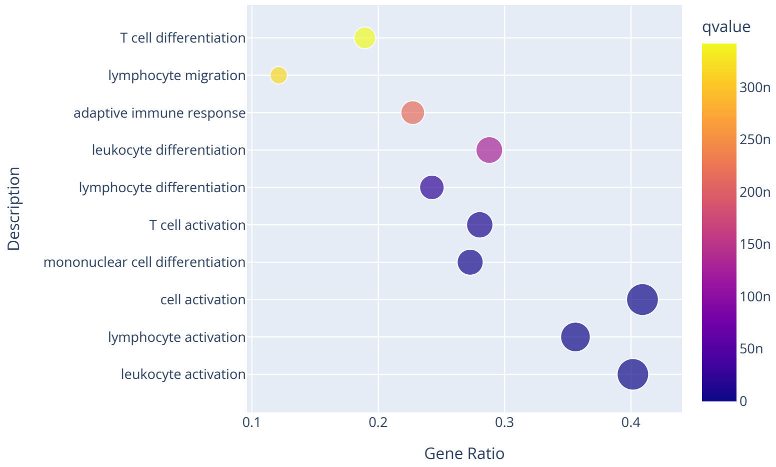

Fig. D14: Dot plot of over-representation analysis (ORA) results for Gene Ontology (GO) Biological Process (BP) terms, based on the differentially expressed genes (DEGs) in the GSE173855 dataset (relapse vs. primary). The x-axis shows the gene ratio (proportion of DEGs in the GO term), the y-axis shows the GO term description, the point size represents the number of DEGs in the term, and the color represents the q-value (FDR-adjusted p-value, where "n" indicates nano, 10^{-9}). The top 10 most significant terms are displayed.

As in previous analyses, *DRaCOoN* was run using three association metrics: Entropy, Pearson's correlation, and Spearman's correlation. The results presented in the main text focus on the entropy-based networks, while results for Pearson and Spearman are provided in the supplementary material. For each association metric, we generated separate networks based on the significance of the differential association metrics: Δr, s, and a combined approach.

Significant interactions were determined based on their FDR-BH adjusted p-values. For the Δr and s networks, a TF-TG interaction was considered differentially regulated if its corresponding adjusted p-value was less than 0.05. For the combined approach, an interaction was deemed significant if either its Δr or its s adjusted p-value was less than 0.05. This allowed us to capture interactions showing significant changes in either the strength or the direction of the association.

In addition to *DRaCOoN*, we evaluated several other differential co-expression analysis methods from the 'dcanr' R package. These methods were applied the same way as explained in D.2.4. Differentially co-expressed gene pairs were identified using an adjusted p-value threshold of 0.05 in agreement with the analysis strategy for this dataset. As shown in Figure D15, the DiffCoEx networks yield the largest networks. Among the *DRaCOoN* Entropy networks, the combined (Δr + s) and *DRaCOoN* Entropy Δr approaches produce larger networks. Other *DRaCOoN* networks, together with *dcanr*'s Entropy, DICER, and z-score, yield no significant networks.

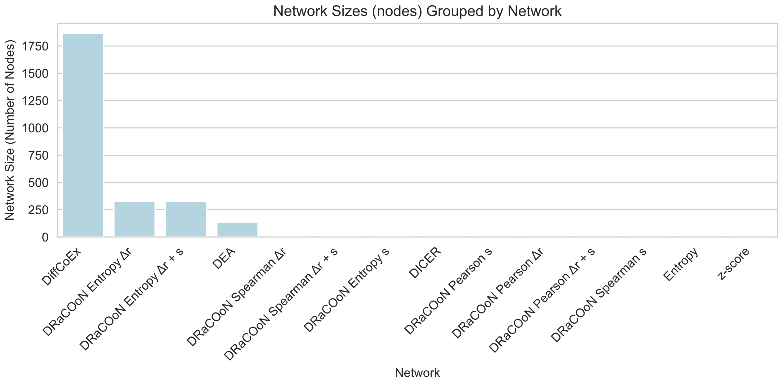

Fig. D15: Network sizes (number of nodes) across different network inference algorithms applied to the GSE173855 (HNSCC) dataset. The bar plot displays the number of nodes included in each network, grouped by algorithm. Bars are sorted in descending order based on network size.

D.3.6. Comparison of Functional Enrichment Profiles: DEG vs. Differential Network

Having performed Over-Representation Analysis (ORA) on both the list of differentially expressed genes (DEGs) derived from standard Differential Expression Analysis (Section D.3.4) and on the set of genes involved in significant differential interactions identified by *DRaCOoN* (using the Entropy $\Delta r + s$ configuration, Section D.3.5), we present a direct comparison of their resulting functional enrichment profiles for the HNSCC GSE173855 dataset in Figure D16. This comparison aims to highlight the potentially distinct biological insights gleaned from feature-level versus interaction-level differential analysis.

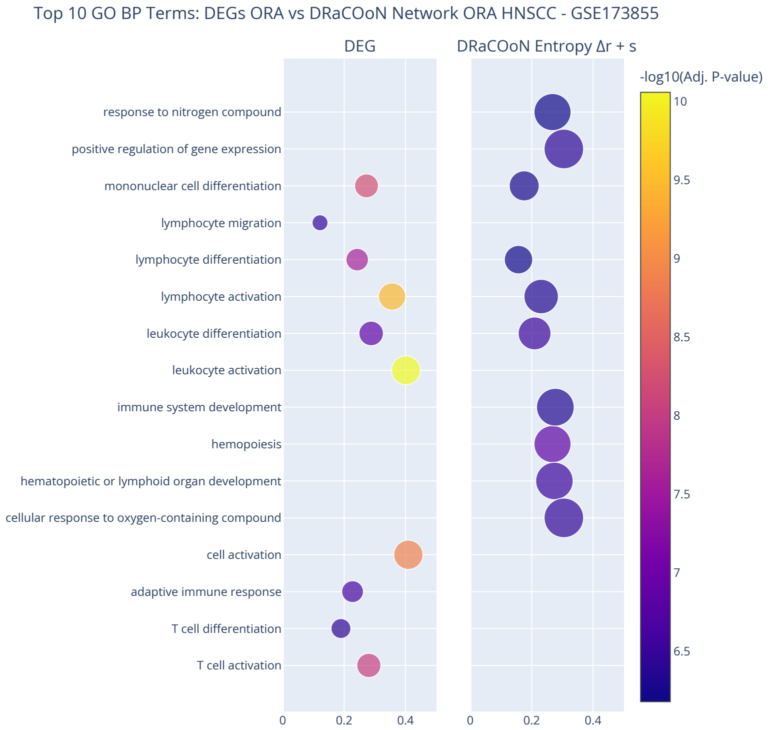

Fig. D16: Comparative Gene Ontology Biological Process (GO BP) enrichment dot plot. Left panel: Top 10 enriched terms for DEGs identified via standard differential expression analysis. Right panel: Top 10 enriched terms for genes involved in significant differential interactions identified by *DRaCOoN* (Entropy $\Delta r + s$ configuration). Analysis performed on the HNSCC GSE173855 dataset (relapse vs. primary). X-axis: Gene Ratio. Y-axis: GO BP Term. Point size: Gene Count. Color: -log10(Adjusted P-value), brighter/higher values indicate greater significance.

Figure D16 reveals notable differences in the biological themes emphasized by the two approaches. While both analyses identify immune-related processes as significant, the differential network analysis (right panel) yields a more focused and consistently stronger enrichment signature centered on immune system development, hematopoietic processes, and lymphocyte/leukocyte activation and differentiation. The significance levels (indicated by color intensity) for these terms are markedly higher compared to the DEG analysis (left panel).

Furthermore, the DRaCOoN results show larger gene ratios and counts (X-axis and point size) for several core immune terms (e.g., "immune system development",

"hematopoietic or lymphoid organ development"), indicating that the network perspective captures a larger, more coordinated group of genes within these pathways compared to simply identifying individually altered genes via DEA. The DEG analysis, while capturing some overlap in immune terms, also highlights processes like "response to nitrogen compound" and "positive regulation of gene expression" which are less prominent or absent in the top network-derived enrichments.

This direct comparison underscores the complementary nature of the analyses. Standard DEA identifies genes with individually significant expression changes, leading to a broader functional profile. In contrast, pathway-level differential network analysis, particularly with the *DRaCOoN* Entropy $\Delta r + s$ method, appears more adept at uncovering statistically robust, coordinated shifts within specific biological systems, such as the immune response pathways strongly implicated in HNSCC recurrence by this analysis.
